# The m^6^A reader IGF2BP2 promotes hepatocellular carcinoma progression via enhancing RELB stability

**DOI:** 10.1186/s43556-026-00465-w

**Published:** 2026-05-07

**Authors:** Hehua Ma, Yuxin Hong, Zhi Xu, Zuyi Weng, Yuanxun Yang, Wei Song, Juan Li

**Affiliations:** 1 Phase I Clinical Trials Unit, Nanjing Drum Tower Hospital, Affiliated Hospital of Medical School, Nanjing University, Nanjing, 210008 China; 2 Phase I Clinical Trials Unit, Nanjing Drum Tower Hospital Clinical College of Nanjing University of Chinese Medicine, Nanjing, 210023 China

**Keywords:** Hepatocellular carcinoma, IGF2BP2, RELB, NF-κB, RNA stability

## Abstract

N^6^-methyladenosine (m^6^A) modifications play a vital role in hepatocellular carcinoma (HCC) progression. However, the function of m^6^A reader proteins in HCC remains poorly understood. Here, we elucidate the role and mechanism of insulin-like growth factor 2 mRNA-binding protein 2 (IGF2BP2) in HCC. In here, we analyzed IGF2BP2 expression in HCC using bioinformatics and clinical samples. The functional role of the IGF2BP2-RELB regulatory axis in HCC progression was assessed through cytological assays and a xenograft HCC mouse model. RNA sequencing, Western blotting, Actinomycin-D assays, and RNA immunoprecipitation (RIP) and methylated RNA immunoprecipitation (MeRIP) assays were performed to investigate the regulatory mechanisms of IGF2BP2 on RELB expression. We found high expression of IGF2BP2 in HCC was positively correlated with poor prognosis. Gain- and loss-of-function assays demonstrated that IGF2BP2 was essential for HCC cell proliferation and migration. IGF2BP2 directly bound to RELB mRNA and enhanced its stability via the KH3/4 domain. Upregulated RELB promoted nuclear translocation of the RELB:p52 dimer, leading to activation of the NF-κB signaling pathway. Furthermore, inhibition of IGF2BP2 and RELB suppressed HCC tumor progression both in vitro and in vivo. Our study demonstrates that IGF2BP2 plays a critical role in HCC progression by stabilizing RELB mRNA and activating the NF-κB signaling pathway. The results suggest that IGF2BP2 may be a potential therapeutic strategy for HCC.

**Supplementary Information:**

The online version contains supplementary material available at 10.1186/s43556-026-00465-w.

## Introduction

Hepatocellular carcinoma (HCC) is the sixth most common malignancy and the third leading cause of cancer-related mortality [1]. Recent advances in systemic therapies have significantly improved the prognosis of HCC patients [2, 3]. However, most patients are diagnosed at advanced stages due to rapid disease progression and the lack of reliable biomarkers for early detection. Consequently, the prognosis remains poor, with a five-year survival rate not exceeding 12% [4]. Therefore, identifying novel therapeutic targets is crucial for improving patient outcomes.

Tumor development involves a series of genetic and epigenetic alterations [5, 6]. N6-methyladenosine (m^6^A) is the most prevalent RNA modification in mammalian cells, accounting for approximately 0.1–0.4% of adenosine residues modification [7]. m^6^A modification is dynamically regulated by m^6^A methylase (METTL3/14 and WTAP), m^6^A demethylase (ALKBH5 and FTO) and m^6^A reader proteins (YTHDF1/2/3, YTHDC1/2, and IGF2BPs) [8, 9]. Accumulating evidence demonstrates that m^6^A modification plays critical roles in transcription, splicing, translation, mRNA stability, and degradation, thereby influencing both physiological processes and oncogenic transformation, including HCC progression [10, 11]. The effects of m^6^A modification are mediated by the reader proteins that recognize methylated sites and regulate gene expression [12, 13]. The insulin-like growth factor 2 mRNA-binding proteins (IGF2BPs), including IGF2BP1/2/3, are newly identified m^6^A reader proteins [14]. Structurally, they contain six RNA-binding domains: two RNA recognition motifs (RRMs) and four K homology (KH) domains [14, 15]. IGF2BPs stabilize target RNAs by recruiting mRNA stabilizers such as HuR and MATR3, thereby promoting RNA storage, localization, and translation [16]. Dysregulation of IGF2BPs, particularly IGF2BP2, has been observed in various malignancies [17, 18]. For instance, upregulated IGF2BP2 enhances glutamine metabolism by regulating MYC, GPT2, and SLC1A5 to promote acute myeloid leukemia (AML) progression [19]. IGF2BP2-mediated CSF2 expression drives malignant transformation of mesenchymal stem cells of gastric cancer [20]. However, the function of IGF2BP2 in HCC has been poorly defined.

Recent studies suggest that m^6^A methylation contributes to tumor progression by regulating downstream oncogenic pathways, including the NF-κB signaling pathway [21]. NF-κB, a redox-sensitive transcription factor, plays a pivotal role in tumor development [22, 23]. In general, NF-κB activation is mediated by the canonical pathway and the non-canonical pathway. The non-canonical NF-κB pathway is activated by processing RELB:p100 into a RELB:p52 heterodimers [24]. The function of the non-canonical NF-κB pathway in cancer progression has been increasingly highlighted [25]. RELB is an important component of the non-canonical NF-κB pathway, and has been proved to participate in tumor progression, including breast cancer [22], prostate cancer [24], and diffuse large B-cell lymphoma (DLBCL) [26]. However, the role of non-canonical NF-κB pathway in HCC progression and its potential interaction with m^6^A modification is still unclear.

In this study, we aimed to study the functions and oncogenic roles of IGF2BP2 and RELB in HCC. We found that IGF2BP2 may enhance the stability of RELB mRNA and the level of protein through its RNA-binding domain. Our findings highlight the critical role of IGF2BP2 in HCC progression and extend a novel mechanism of m^6^A methylation-mediated NF-κB signaling in the pathogenesis of HCC.

## Results

### IGF2BP2 is upregulated in hepatic tissue of HCC patients and positively associated with poor prognosis

**Fig. 1:**
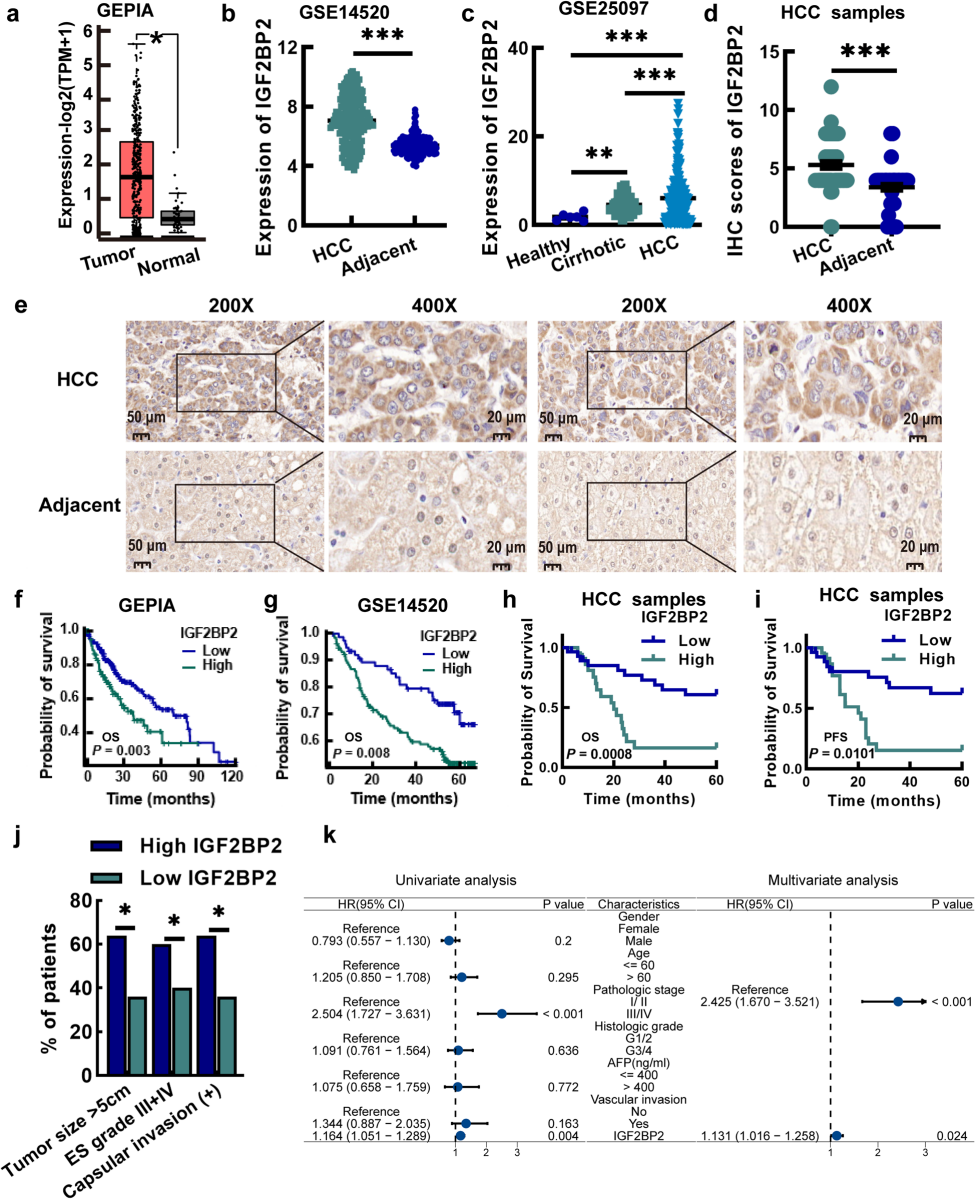
IGF2BP2 is upregulated and correlates with poor prognosis in HCC. **a** Expression of IGF2BP2 in HCC tissues (*n* = 369) and normal liver tissues (*n* = 50) based on GEPIA dataset. **b** The expression of IGF2BP2 in HCC tissues (*n* = 225) and adjacent non-tumor tissues (*n* = 220) in GSE14520 dataset. **c** The expression of IGF2BP2 in healthy liver tissues (*n* = 6), cirrhotic tissues (*n* = 40) and HCC tissues (*n* = 268) in GSE25097 dataset. **d** IHC scores of IGF2BP2 staining in HCC tissues and adjacent non-tumor tissues (*n* = 50). **e** Representative images of IGF2BP2 IHC staining in HCC tissues and adjacent non-tumor tissues. **f**, **g** Relationship between IGF2BP2 expression and survival prognosis in HCC samples in GEPIA (**f**) and GSE14520 (**g**) dataset. **h**, **i** The overall survival and progression-free survival analysis of 50 HCC patients stratified by IGF2BP2 expression level. **j** Correlation analysis of IGF2BP2 and clinical features of HCC patients. **k** Univariate and multivariate Cox proportional hazards analyses of IGF2BP2 for overall survival in patients with HCC. Data are mean ± SEM values. *P < 0.05, **P < 0.01,***P < 0.001

We analyzed the expression of IGF2BP 1/2/3 genes in HCC utilizing the Gene Expression Profiling Interactive Analysis (GEPIA) datasets. The results revealed that only IGF2BP2 mRNA expression was significantly upregulated, whereas the expression levels of IGF2BP1 and IGF2BP3 showed no statistically significant differences (Fig. 1a and Fig. S1a). The results of multiple Gene Expression Omnibus (GEO) datasets also showed that the expression of IGF2BP2 in HCC tissues was higher than that in adjacent non-tumor tissues (Fig. 1b and Fig. S1b). Notably, IGF2BP2 expression was significantly upregulated in cirrhotic and HCC tissues compared with healthy liver tissues (Fig. 1c). We also found the increased expression of IGF2BP2 in the 50 HCC tissues, compared to that in the 50 adjacent non-tumor tissues (Fig. 1d and e).

Moreover, we analyzed the association of IGF2BP2 level and the overall survival time of the patients based on the data from GEPIA and GEO datasets (Fig. 1f and g), as well as the data in the 50 patients in the present study (Fig. 1h and i). The results showed that those with higher IGF2BP2 expression had shorter survival time (Fig. 1f to i). Further analysis of the 50 HCC patients in this study showed the higher IGF2BP2, the worse progression-free survival (Fig. 1i). The patients with the larger tumor tissue, III/IV Edmondson Steiner grade, or capsular invasion had higher IGF2BP2 expression (Fig. 1j). Furthermore, univariate and multivariate Cox proportional hazard analysis indicated that high IGF2BP2 expression was associated with poor survival in HCC patients (Fig. 1k).

### IGF2BP2 promotes HCC proliferation and migration

Since IGF2BP2 was increased in the tumor tissue of HCC patients (Fig. 1), we measured the expression of IGF2BP2 in the LO2 (immortalized human hepatic cell line) and established HCC cell lines, Hep3B, HepG2, Huh7 and MHCC97H. The results indicated that the expression level of IGF2BP2 in HCC cells were higher than that in LO2, and between HCC cells, the expression in Hep3B was lowest and in the HepG2 was highest (Fig. S2).

**Fig. 2:**
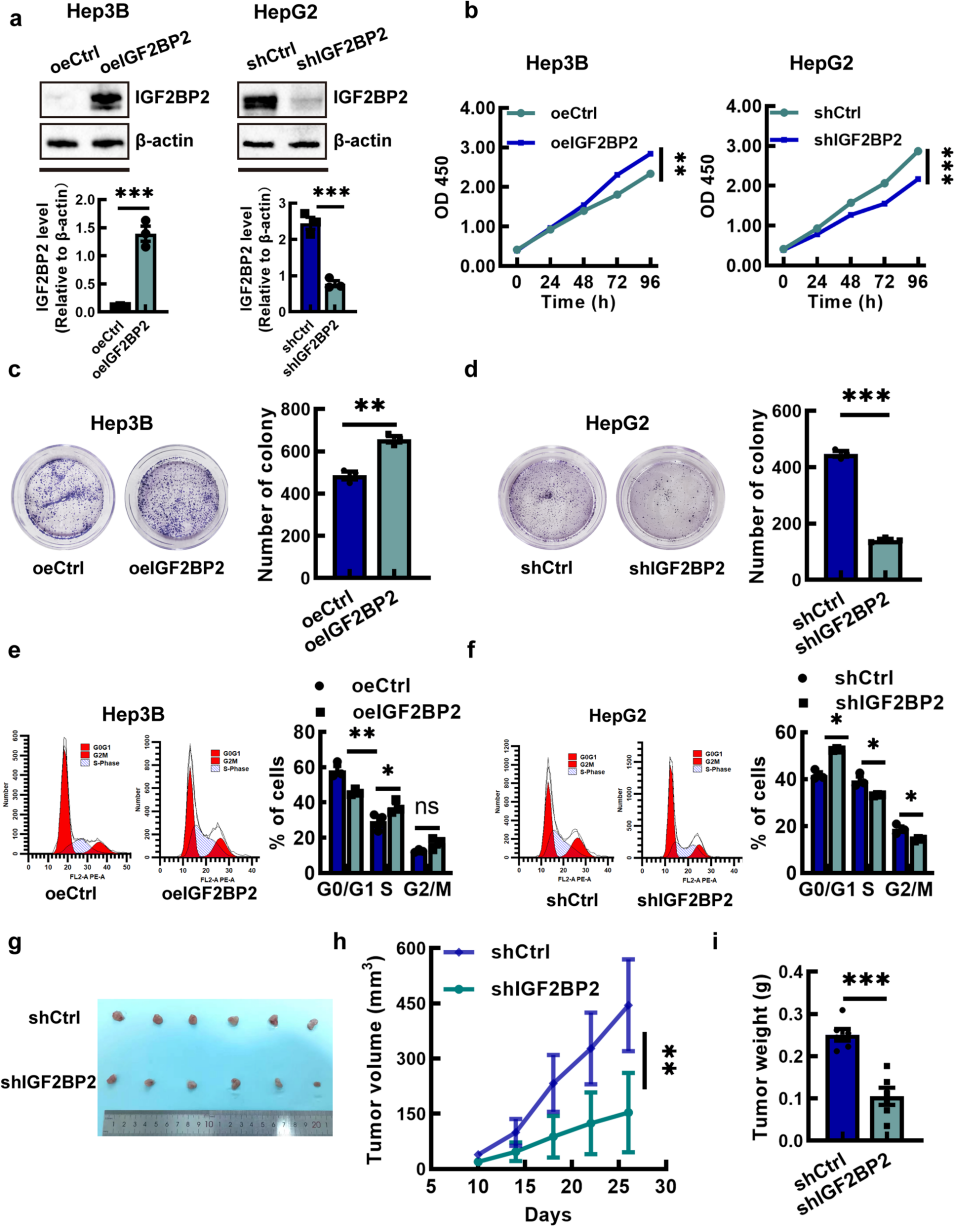
IGF2BP2 promotes HCC proliferation. **a** Western blotting of IGF2BP2 expression in stable IGF2BP2 overexpression and IGF2BP2 knockdown HCC cells. **b** CCK-8 assays of Hep3B with IGF2BP2 overexpression (Hep3B-oeIGF2BP2) and HepG2 with IGF2BP2 knockdown (HepG2-shIGF2BP2). **c**, **d** Colony formation assays of Hep3B-oeIGF2BP2 cells and HepG2-shIGF2BP2 cells. **e**, **f** Cell cycle assays of Hep3B-oeIGF2BP2 cells and HepG2-shIGF2BP2 cells. **g**-**i** Tumor volume and weight of subcutaneous tumor (*n* = 6). Data are mean ± SEM values (*n* = 3). *P < 0.05, **P < 0.01, ***P < 0.001

To elucidate the role of IGF2BP2 in HCC progression, we established stable cell lines by transducing Hep3B cells with lentiviruses encoding IGF2BP2 (Hep3B-oeIGF2BP2) and silencing IGF2BP2 in HepG2 cells using lentiviral shRNA (HepG2-shIGF2BP2). The protein level of IGF2BP2 was verified by Western blotting (Fig. 2a). The results of CCK-8 assay demonstrated that overexpression of IGF2BP2 significantly promoted cell proliferation, while interference of IGF2BP2 expression impeded cell proliferation (Fig. 2b). These findings were further corroborated by the colony formation assays (Fig. 2c and d). Meanwhile, cell cycle assays showed that upregulation of IGF2BP2 accelerated the proliferation process, manifested as a reduction in the G1 phase and an increase in the S phase, whereas inhibition of IGF2BP2 led to more cells arrested in G1 phase (Fig. 2e and f). Moreover, we established an animal HCC model by subcutaneous injection of HepG2 cells in nude mice to validate the effect of IGF2BP2 on the progression of HCC in vivo. The findings of monitoring mice every 3–4 days indicated that interference with IGF2BP2 restrained the growth of xenograft tumor (Fig. 2g), and the tumor volumes and masses in the shIGF2BP2 group were much smaller (Fig. 2h and i), when the mice were euthanized after 4 weeks.

**Fig. 3:**
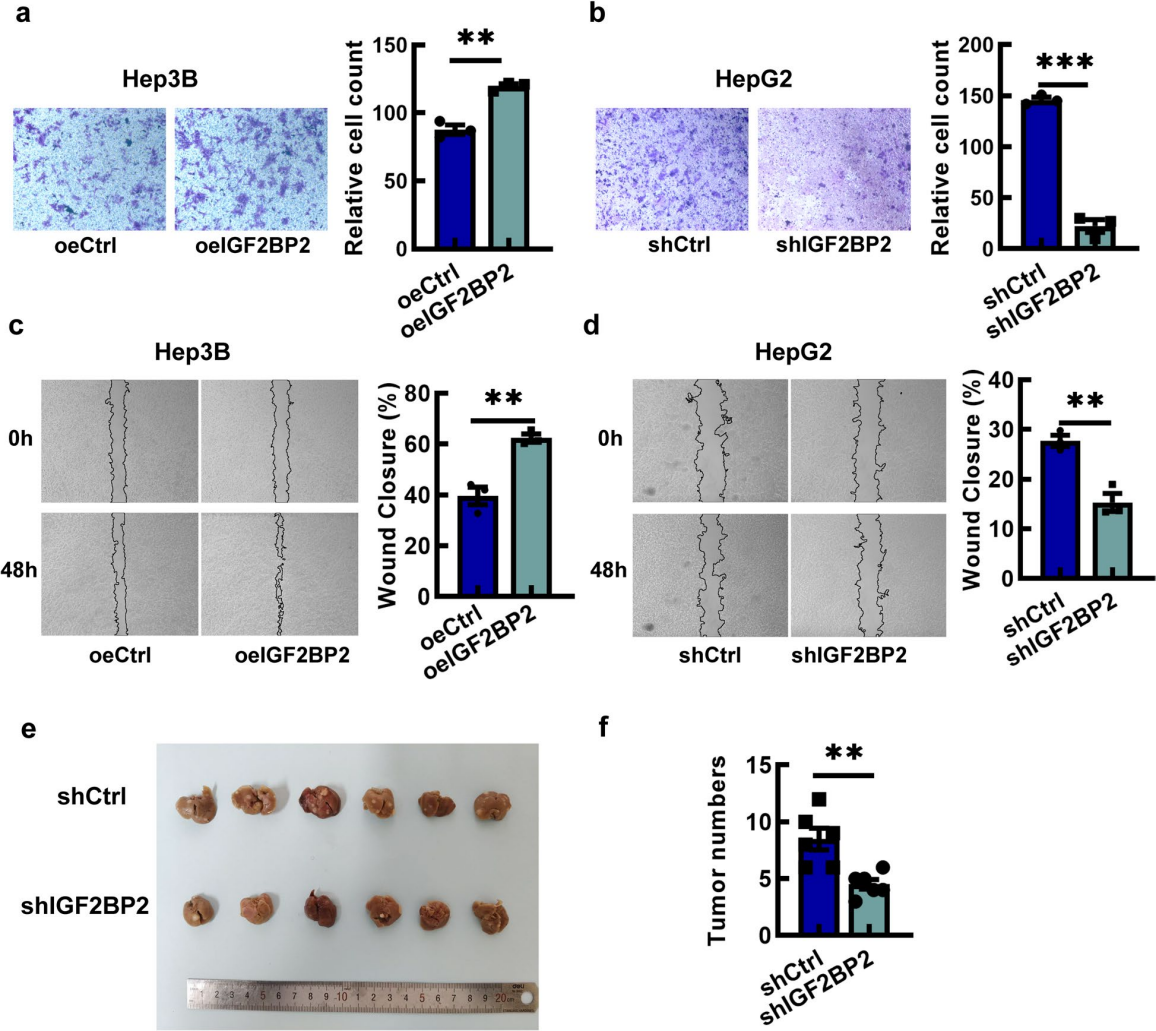
IGF2BP2 promotes HCC migration. **a**, **b** Transwell assays of Hep3B-oeIGF2BP2 cells and HepG2-shIGF2BP2 cells. (Scale bar = 200 μm) **c**, **d** Wound healing assays of Hep3B-oeIGF2BP2 cells and HepG2-shIGF2BP2 cells. (Scale bar = 100 μm). **e**, **f** The knockdown of IGF2BP2 inhibited the liver metastasis nodules (*n* = 6). Data are mean ± SEM values (*n* = 3). **P < 0.01, ***P < 0.001

Transwell assays showed that Hep3B cells with upregulated IGF2BP2 level (oeIGF2BP2 group) migrated significantly more compared to Hep3B cells in the control group (empty-vector treated Hep3B cells) (Fig. 3a), while HepG2 cells with IGF2BP2 knockdown impaired cell migratory capacity (Fig. 3b). The wound healing assay also showed that overexpression of IGF2BP2 resulted in a higher wound healing rate, whereas IGF2BP2 interference decreased wound healing rate (Fig. 3c and d). Subsequently, we established a liver metastatic model by tail vein injections to evaluate the function of IGF2BP2 in promoting HCC metastatic capacity. After injection with shIGF2BP2 cells or control cells for 7 weeks, the mice were euthanized, and liver metastatic nodules were counted. Few metastatic nodules were observed in the livers of the shIGF2BP2 group (Fig. 3e and f).

Furthermore, apoptosis assays showed that IGF2BP2 knockdown markedly increased the apoptosis of HCC cells, in contrast, IGF2BP2 overexpression led to a significant reduction of apoptotic cells (Fig. S3a and 3b).

### RELB is a possible downstream target of IGF2BP2

**Fig. 4:**
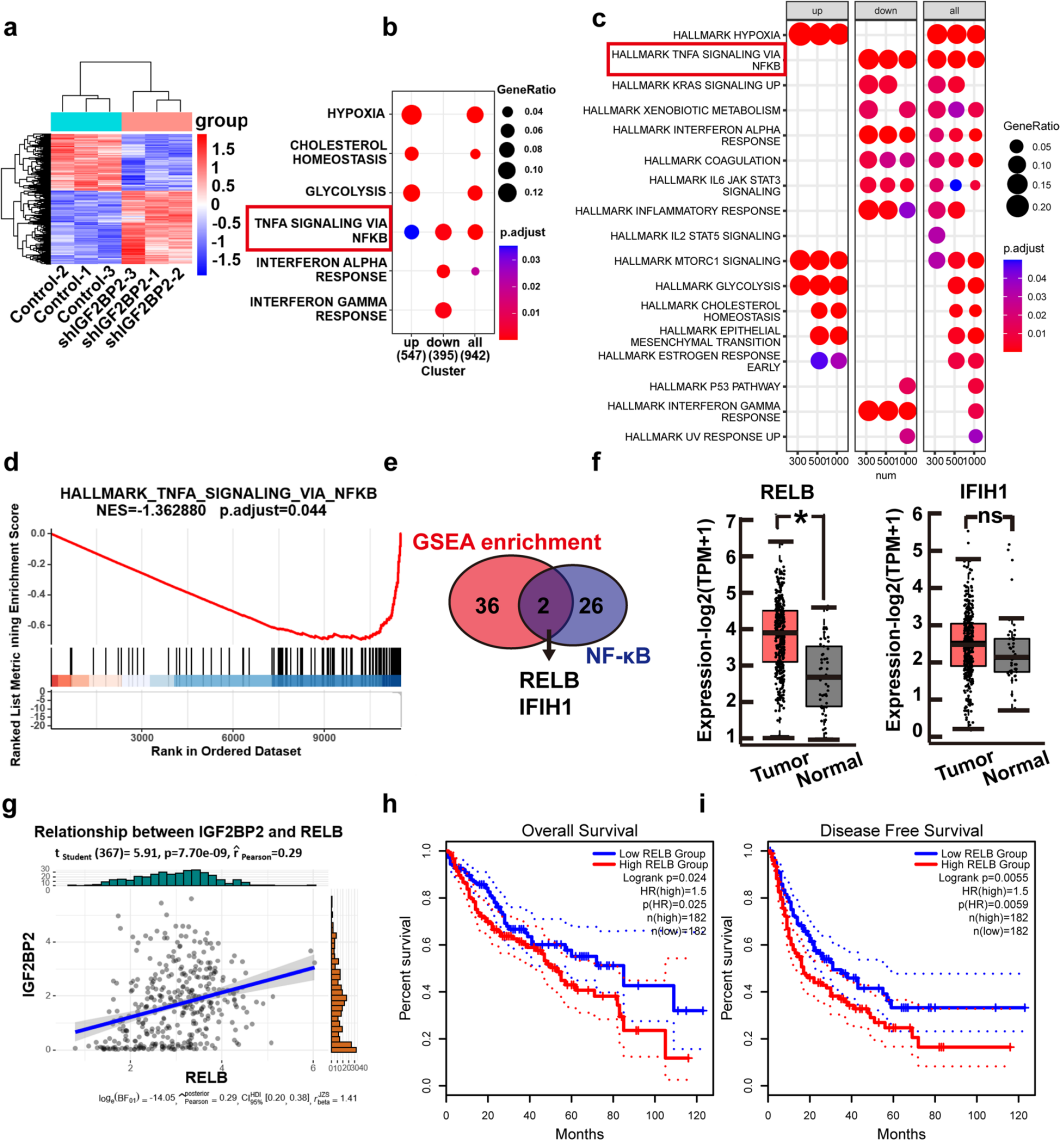
RELB was a potential target of IGF2BP2. **a** Heatmap showing the profiles of transcripts after knockdown IGF2BP2 in HepG2 cells. **b** KEGG pathway enrichment analysis of the upregulated, downregulated and all differently expressed genes. **c** KEGG pathway enrichment analysis of different number of downregulated genes. **d** GSEA analysis of all downregulated genes. **e** Venn diagram depicting the overlapped genes between GSEA enriched genes and key genes of NF-κB signaling pathway. **f** The expression of RELB and IFIH1 in GEPIA dataset. **g** Correlation analysis of IGF2BP2 and RELB in GEPIA dataset. **h**, **i** Overall survival and disease-free survival analysis of HCC patient in GEPIA dataset between RELB-low group and RELB-high group. Data are mean ± SEM values (*n* = 3). *P < 0.05, ns, not significant

Having demonstrated the effect of IGF2BP2 on the progression of HCC, we performed RNA-seq sequencing by extracting total RNA from HepG2-shIGF2BP2 cells (inhibited expression of IGF2BP2) to search for the target of IGF2BP2. In total, 942 differentially expressed genes were found, of which 395 genes were downregulated and 547 genes were upregulated (Fig. 4a). These genes were analyzed based on the Kyoto Encyclopedia of Genes and Genomes (KEGG), and it was revealed that the downregulated genes were significantly enriched in the NF-κB signaling pathway (Fig. 4b). Further analysis of different numbers of downregulated genes in KEGG also showed a significant enrichment in the NF-κB signaling pathway (Fig. 4c). Moreover, gene set enrichment analysis (GSEA) also indicated that the downregulated genes were mainly enriched in the NF-κB signaling pathway (Fig. 4d). Therefore, we hypothesized that IGF2BP2 may take part in the NF-κB signaling pathway. We searched the overlapped genes in the downregulated genes revealed in our study with the genes in the NF-κB signaling pathway in GSEA and identified two genes, RELB and IFIH1, overlapped (Fig. 4e).

Analysis of the GEPIA dataset found that the expression of RELB was significantly upregulated in HCC, yet the expression of IFIH1 was not remarkably upregulated (Fig. 4f). Correlation analysis of the GEPIA dataset showed that the expression level of IGF2BP2 was positively associated with that of RELB (Fig. 4g). Based on the GEPIA dataset, HCC patients with the elevated expression of RELB had lower survival time (Fig. 4h) and a lower rate of tumor-free survival (Fig. 4i). Therefore, we hypothesized that RELB may be a possible downstream target of IGF2BP2.

### IGF2BP2 facilitates RELB expression in an m^6^A dependent manner

**Fig. 5:**
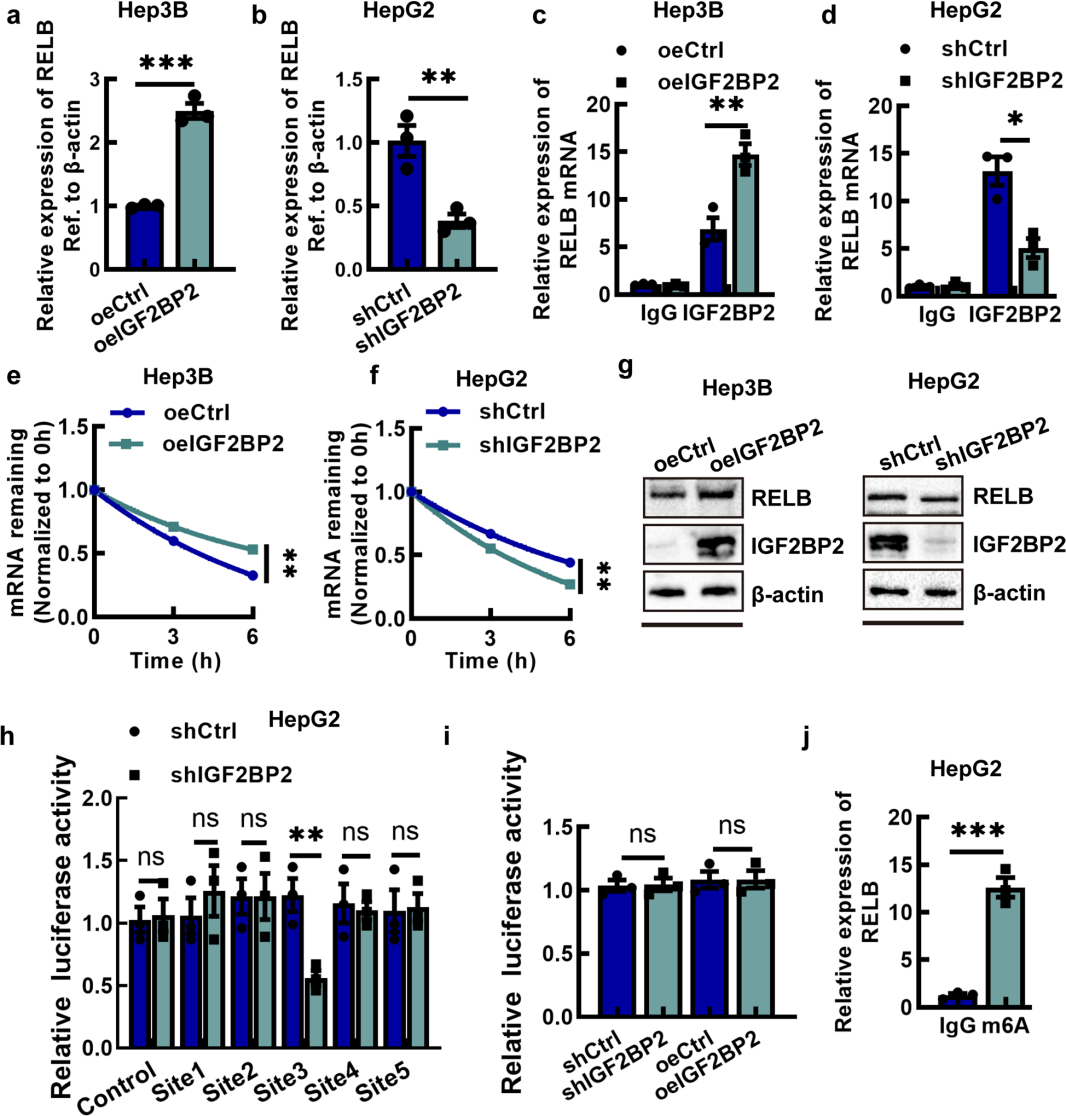
IGF2BP2 facilitates RELB expression in an m^6^A dependent manner. **a**, **b** The mRNA level of RELB in Hep3B-oeIGF2BP2 cells and HepG2-shIGF2BP2 cells. **c**, **d** RIP-qPCR analysis of the interaction between RELB mRNA and IGF2BP2 protein in oeIGF2BP2 and shIGF2BP2 cells using a IGF2BP2 antibody. **e**, **f** The study of RELB mRNA stability in Hep3B-oeIGF2BP2 cells and HepG2-shIGF2BP2 cells treated with Actinomycin D for 0, 3 and 6 h. **g** The protein level of RELB in Hep3B-oeIGF2BP2 cells and HepG2-shIGF2BP2 cells. **h** The dual‐luciferase reporter assays on HepG2-shCtrl and shIGF2BP2 cells. **i** The dual‐luciferase reporter assays on HepG2-shIGF2BP2 and Hep3B-oeIGF2BP2 cells transfected with luciferase reporter gene vectors with site 3 mutant sequence. **j** MeRIP‐PCR of m^6^A enrichment of site 3 on RELB mRNA. Data are mean ± SEM values (*n* = 3). *P < 0.05, **P < 0.01, ***P < 0.001, ns, not significant

To clarify whether RELB may be a target of IGF2BP2, we performed qPCR to measure the mRNA levels of RELB in Hep3B-oeIGF2BP2 and HepG2-shIGF2BP2 as well as in empty-vector treated Hep3B and HepG2, and found that the mRNA levels of IGF2BP2 were positively correlated with those of RELB (Fig. 5a and b). RIP-qPCR showed that IGF2BP2 co-precipitated RELB mRNA in HCC cells (Fig. 5c and d). The RNA stability assay manifested that the RELB mRNA in Hep3B-oeIGF2BP2 was significantly less degraded, and the mRNA in HepG2-shIGF2BP2 was much more degraded (Fig. 5e and f). Similarly, Western blotting showed that IGF2BP2 up-regulated RELB protein levels (Fig. 5g and h, and Fig. S4a and S4b). To further confirm that IGF2BP2 binds to RELB mRNA via m⁶A modification, we first predicted the potential binding m⁶A modification sites on RELB mRNA using the SCRAMP tools and five m^6^A sites were ultimately determined (Fig. S5). The sequences corresponding to the aforementioned predicted binding sites were inserted into the Pgl3 vector carrying the firefly luciferase reporter, and dual-luciferase reporter assays were subsequently conducted in both HepG2-shCtrl and HepG2-shIGF2BP2 cell lines (Fig. 5h). The results showed that the luciferase activity of the reporters containing site 3 sequences were reduced in the IGF2BP2 knockdown group. We then mutated the key m^6^A modification of site 3 and reconstructed the firefly luciferase reporter gene vectors for transfection. The results showed that the luciferase activity of the reporters containing mutant sequences were similar between the IGF2BP2 knockdown group and control group (Fig. 5i). MeRIP‐PCR verified that the key recognition sequences of the site 3 had indeed m^6^A modifications (Fig. 5j). Besides, we analyzed RELB expression in HCC cells transfected with siRNA targeting IGF2BP1 or IGF2BP3. The results showed that RELB expression exhibited no significant change upon IGF2BP1 or IGF2BP3 knockdown. (Fig. S6).

**Fig. 6:**
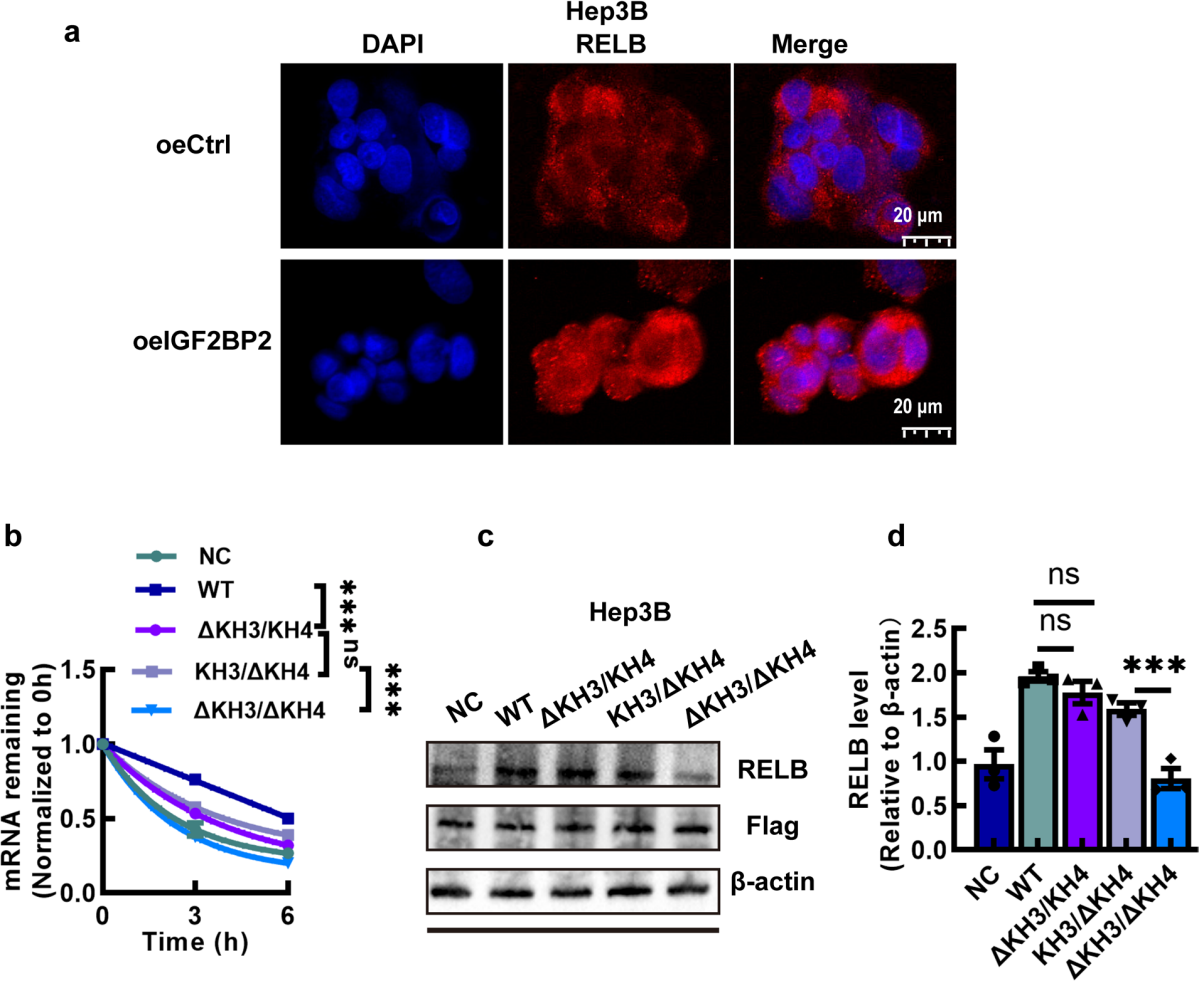
IGF2BP2 facilitates RELB expression via KH3/4 domain. **a** The immunofluorescence assay of RELB:p52 dimer nuclear translocation in Hep3B-oeIGF2BP2. **b**The decay rate of RELB mRNA in Hep3B cells transfected with the IGF2BP2 WT plasmid, KH3 (ΔKH3/KH4) or KH4 (KH3/ΔKH4) single mutant plasmid or KH3/4 double mutants plasmid (ΔKH3/ΔKH4) after treated with Actinomycin D for the indicated times. **c**, **d** Western blotting of RELB protein level in Hep3B cells with IGF2BP2 WT, KH3, KH4 or KH3/4 mutant. Data are mean ± SEM values (*n* = 3). *P < 0.05, **P < 0.01, ***P < 0.001, ns, not significant

Furthermore, to verify that IGF2BP2 participates in the NF-κB pathway via RELB, we performed immunofluorescence assays. The results showed RELB protein in the nuclei of Hep3B-oeIGF2BP2 was more than that in the nuclei of empty-vector treated Hep3B in immunofluorescence microscopy (Fig. 6a), whereas the RELB protein in the nuclei of HepG2-shIGF2BP2 was less than that in the nuclei of empty-vector treated HepG2 (Fig. S7a). Moreover, RELB protein in the nuclei of Hep3B-siRELB was less than that in the nuclei of Hep3B-siCtrl (Fig. S7b), which showed that the inhibition of RELB blocked the nuclear translocation of RELB:p52 dimer. These results indicated IGF2BP2 participates NF-κB pathway though binding the m^6^A modification site of RELB and regulating its protein expression.

Since GXXG motif within the KH3 and KH4 RNA-binding domains of IGF2BP2 is critical for m^6^A methylation recognition [14, 27], we tried to characterize the functional role of this conserved motif in the effect of IGF2BP2 on HCC. We generated site-directed mutants of the KH3 and KH4 and the combination of these two mutants through targeted mutagenesis of the GXXG motif to GDDG. After transfection of Hep3B cells with each of these engineered constructs, single KH3 or KH4 domain mutation significantly reduced RELB mRNA stability, and the combination of the mutants more potently reduced RELB mRNA stability in the RNA stability assays (Fig. 6b). Western blotting displayed the corresponding reduction in RELB protein level in HCC cells harboring the KH3/KH4 double mutation (Fig. 6c and d).

### RELB promotes HCC progression

To elucidate the effect of RELB in HCC progression, we disrupted RELB expression by siRNA in Hep3B and HepG2 cells. The expression of RELB was verified by Western blotting (Fig. S8a). The results of CCK-8 assays (Fig. S8b) and colony formation assays (Fig. S8c and S8d) showed that inhibition of RELB expression suppressed the proliferation of HCC cells. Furthermore, cell cycle analysis by flow cytometry revealed that the RELB-silenced group (siRELB) contained significantly fewer cells in the S phase and a marked increased cells in G1 phase, compared to non-targeting siRNA controls (Fig. S8e and S8f). In Transwell assays, the number of migrated siRELB cells was reduced, compared to that in control group (Fig. S8g and S8h). In wound healing assays, siRELB cells exhibited a decrease in scratch closure velocity at 48 h, compared to the control group (Fig. S9a and S9b). Thus, RELB silencing significantly impaired cell migration. Furthermore, apoptosis assays showed that RELB knockdown markedly reduced the apoptosis of HCC cells (Fig. S9c and S9d).

### Interference with RELB can reverse the carcinogenic effect of IGF2BP2

**Fig. 7:**
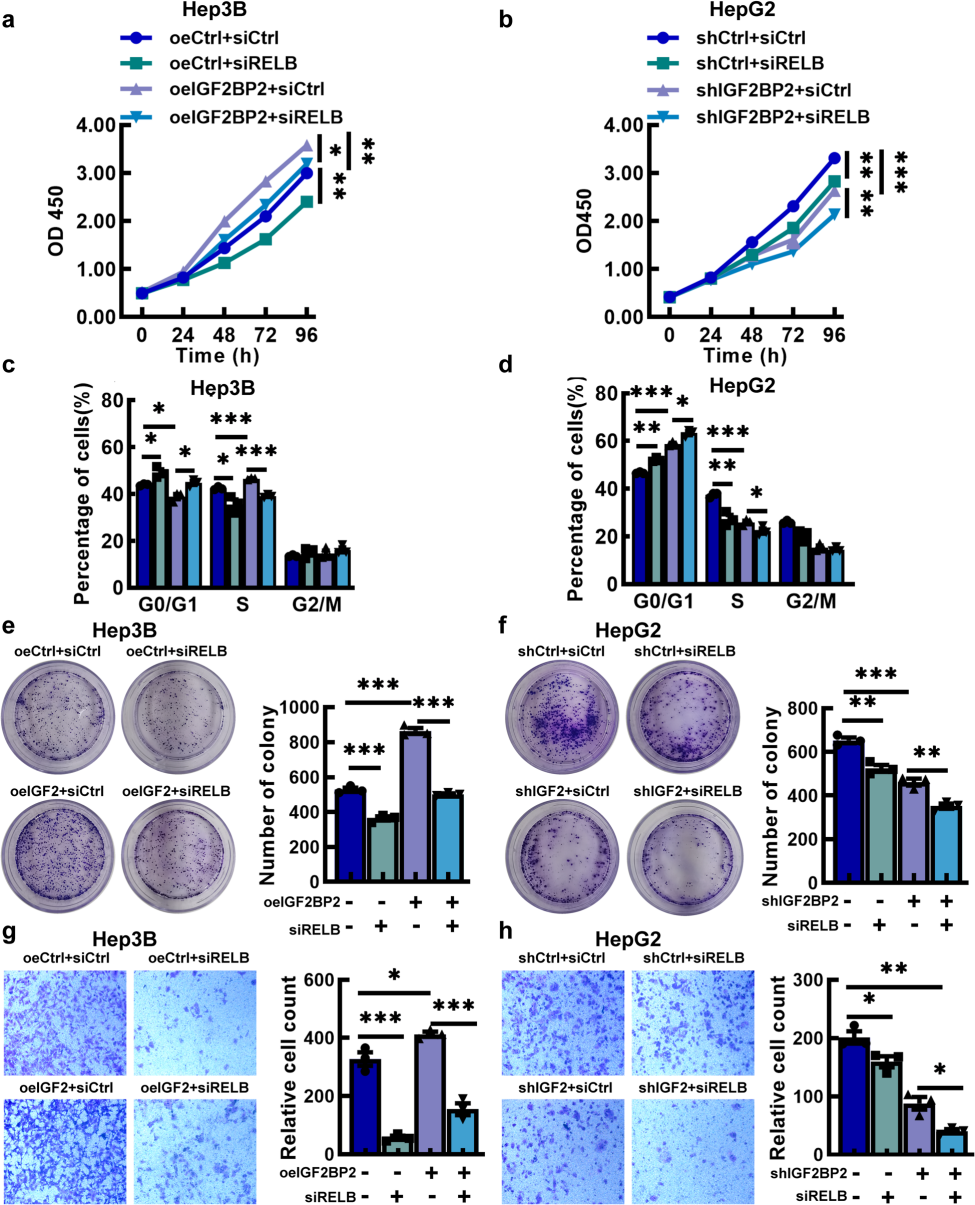
Interference with RELB can reverse the carcinogenic effect of IGF2BP2. **a**, **b** CCK-8 assays of Hep3B-oeIGF2BP2 cells and HepG2-shIGF2BP2 cells with silencing RELB (siRELB). **c**, **d** Cell cycle assays in Hep3B-oeIGF2BP2 cells and HepG2-shIGF2BP2 cells with siRELB. **e**, **f** Colony formation assays in Hep3B-oeIGF2BP2 cells and HepG2-shIGF2BP2 cells with siRELB. **g**, **h** Cell migration assays of Hep3B-oeIGF2BP2 cells and HepG2-shIGF2BP2 cells with siRELB. (Scale bar = 200 μm) Data are mean ± SEM values (*n* = 3). *P < 0.05, **P < 0.01

To further validate the dependency of IGF2BP2-mediated HCC progression on RELB, rescue assays were performed by silencing RELB (siRELB) in Hep3B-oeIGF2BP2 and HepG2-shIGF2BP2 cells using small interfering RNA. As controls, cells were transfected with non-targeting siRNA. CCK-8 assays (Fig. 7a) and colony formation assays (Fig. 7e) demonstrated that the promoting proliferative capacity of IGF2BP2 was significantly counteracted by silencing of RELB. The combined inhibition of IGF2BP2 and RELB exhibited a more pronounced inhibition on cell proliferation (Fig. 7b and f). Silencing of RELB significantly inhibited IGF2BP2-mediated cell cycle progression from G1 to S phase, as evidenced by reduced proportions of cells in the S phase and increased proportions of cells in G1 phase (Fig. 7c). Conversely, combined knockdown of IGF2BP2 and RELB (shIGF2BP2 + siRELB) synergistically decreased the cell cycle progression, with a marked accumulation of cells in the G1 phase, compared to single-knockdown groups (Fig. 7d). Depletion of RELB (siRELB) significantly inhibited the IGF2BP2-overexpression (oeIGF2BP2)-induced enhancement of cell migration, as the number of migrated cells in Transwell was significantly reduced, compared to that in oeIGF2BP2 group in which RELB was not depleted (Fig. 7g). Moreover, combined depletion of IGF2BP2 and RELB (siRELB + shIGF2BP2) acted synergistically to further suppress cell migration, with a greater reduction in migrated cells compared to either single knockdown (shIGF2BP2 + siCtrl and shCtrl + siRELB) or the negative control (shCtrl + siCtrl) (Fig. 7h). These results were validated by wound healing assays (Fig. S10a and S10b). Moreover, the overexpression of RELB could reverse IGF2BP2 knockdown-induced suppression of HCC cell proliferation (Fig. S10c).

###  Combined inhibition of RELB and IGF2BP2 can delay the progression of HCC ex vivo and in vivo

**Fig. 8:**
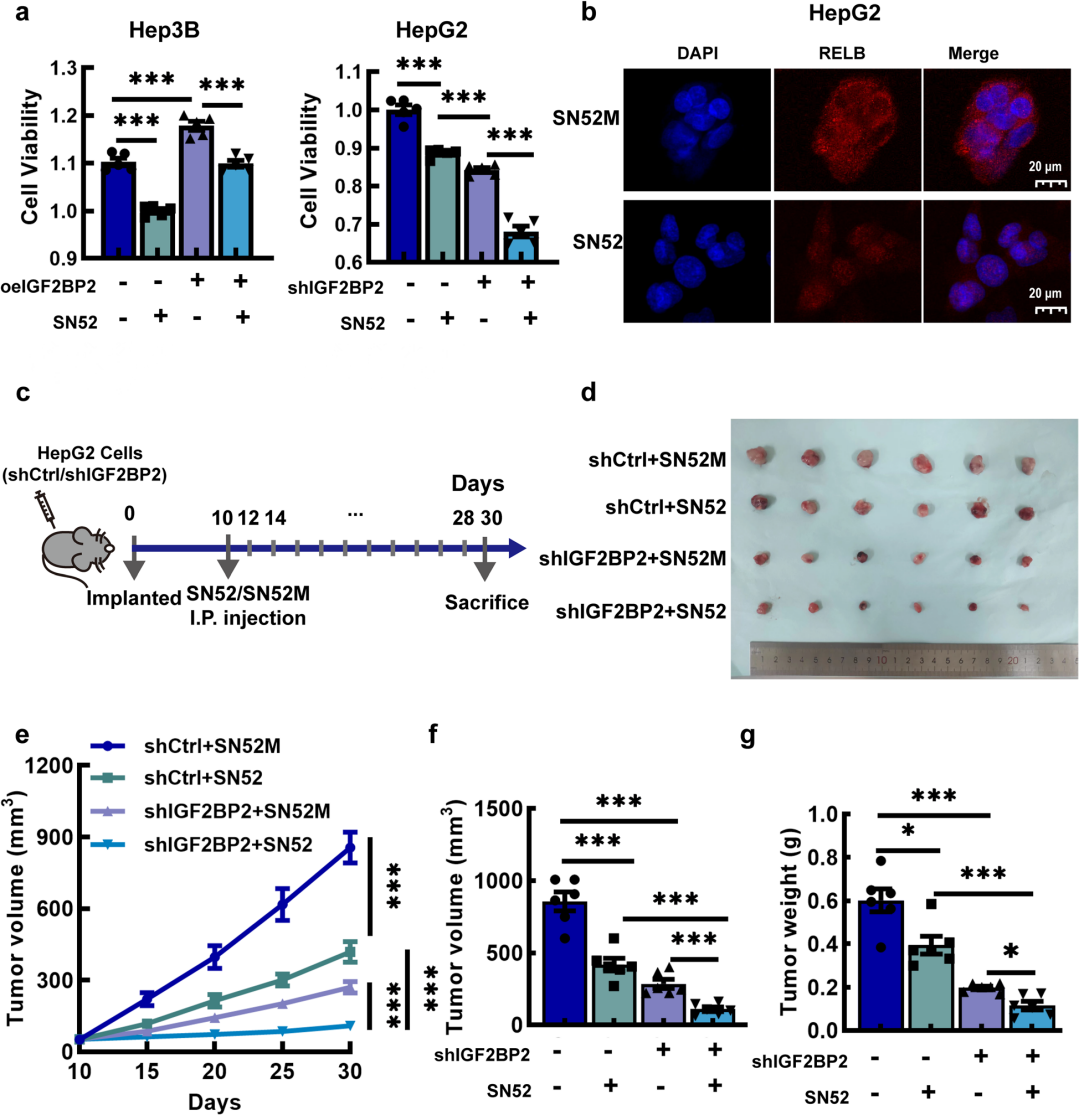
Combined inhibition of RELB and IGF2BP2 can inhibit HCC progression. **a** The effect of SN52 in HCC cells with overexpression IGF2BP2 and knockdown IGF2BP2. **b** The effect of SN52 on the nuclear translocation of the RELB:p52 dimer in HepG2 cells. **c** The schematic of therapeutic role of SN52 combined with IGF2BP2 inhibition was evaluated with subcutaneous xenograft implantation model. **d** Images of subcutaneous tumor (*n* = 6). **e** Growth of subcutaneous tumor as described in c. **f**-**g** Tumor volume and weight of subcutaneous tumor (*n* = 6). Data are mean ± SEM values (*n* = 3). *P < 0.05, ***P < 0.001

To determine if RELB is a critical mediator downstream of IGF2BP2, we treated HCC cells with SN52, which is a specific inhibitor of RELB, and may inhibit non-canonical NF-κB signaling pathway through blocking nuclear translocation of RELB:p52 dimer [28]. The results showed that SN52 restricted the cell proliferation, especially the HCC cells with downregulated IGF2BP2 (Fig. 8a), suggesting that combined inhibition of RELB and IGF2BP2 may impede the progression of HCC. Immunofluorescence assays also confirmed that treatment with SN52 inhibited the nuclear translocation of the RELB:p52 dimer (Fig. 8b).

Furthermore, we implanted HepG2-shIGF2BP2 cells and empty-vector treated HepG2 cells into nude mice to verify the inhibition of HCC progression by depleting RELB and IGF2BP2. The mice with xenograft tumor firstly injected intraperitoneally with SN52 (40 μg/mouse/time) on day 10 after tumor cell implantation and then injected with same SN52 dose every other day (Fig. 8c). The results showed that tumor growth rates were significantly lower in shIGF2BP2 + SN52 groups compared with those in the controls (Fig. 8d and e). By day 30, shIGF2BP2 + SN52 combination group showed the greatest reduction in mean tumor volume (Fig. 8d). The mean tumor volume in the shIGF2BP2 + SN52 group was 108.58 ± 15.74 mm3, significantly lower that the volume of 856.3 ± 64.65 mm3 in shCtrl + SN52M group (*P* < 0.001, 87% reduction) (Fig. 8f). Similarly, the tumor weight in the shIGF2BP2 + SN52 group was significantly lower than that in the shCtrl + SN52M group (0.12 ± 0.02 vs 0.60 ± 0.05 g, *P* < 0.001) (Fig. 8g).

## Discussion

In this study, we observed that IGF2BP2 was upregulated in HCC tissues and IGF2BP2 enhanced the proliferation and migration of HCC cells. We revealed that IGF2BP2 stabilized RELB mRNA through its m^6^A reader enzymatically active domains (KH3/4), followed by enhanced activation of the non-canonical NF-κB signaling pathway by RELB. Therefore, we demonstrate that IGF2BP2 and the RELB-mediated NF-κB pathway are functionally involved in HCC development. Furthermore, we found that suppression of IGF2BP2 effectively inhibited HCC progression in cell cultures and nude mice. The results indicate that IGF2BP2 may be a potential target for HCC treatment.

Recent studies have underscored the role of IGF2BP2 in other malignancies [29]. For instance, IGF2BP2 upregulates SGMS2 expression to promote sphingomyelin synthesis, which facilitates PD-L1 localization on membrane lipid rafts and enhances immune evasion in pancreatic adenocarcinoma [30]. In T-cell acute lymphoblastic leukemia, IGF2BP2 directly binds to the oncogene NOTCH1 to enhance its stabilization, thereby contributing to T-cell leukemogenesis [15]. Consistent with these findings, our analysis of GEPIA and GEO datasets, along with an independent HCC cohort, revealed IGF2BP2 overexpression that strongly correlated with poor prognosis. A series of experiments showed that IGF2BP2 may promote proliferation and migration of HCC cells, and interference of IGF2BP2 may attenuate these malignant phenotypes and moreover, suppress tumor growth in vivo (Figs. 2, and 3). These findings indicate IGF2BP2 modulates the expression of oncoproteins and alters HCC cell behavior, thereby driving cancer progression. Furthermore, IGF2BP proteins have been reported to be functionally redundant. We analyzed the expression of IGF2BP1 and IGF2BP3 in the GEPIA database and found that although their expression exhibited an upward trend, no statistically significant difference was observed (Fig. S1a).

In the present study, we identified RELB as a downstream target of IGF2BP2, which may be associated with the molecular mechanisms underlying IGF2BP2-mediated progression of HCC. It has shown that IGF2BP2 directly recognized and bound to the m^6^A site on FEN1 mRNA and TRPC7-AS1 to enhance their mRNA stability by exercising the function of reading proteins [31, 32]. Considering the function of IGF2BP2, we conducted Dual‐luciferase reporter assays and MeRIP-qPCR assay to identify the specific m^6^A sites on RELB mRNA that are recognized by IGF2BP2(Fig. 5). RELB is a key component of the NF-κB signaling pathway. The role of the RELB-dependent non-canonical pathway in tumor progression has garnered increasing attention [33]. RELB, as a key member of the NF-κB pathway, plays a critical role in the malignant progression of various cancers [24, 34]. In our study, we also validated that IGF2BP2 directly bound to RELB mRNA, thereby stabilizing RELB transcripts and increasing RELB protein levels as well as its nuclear translocation (Figs. 5 and 6). And RELB knockdown suppresses the nuclear translocation of RELB:p52 dimer, which has been verified involved in RELB-activated noncanonical NF-κB pathway [22]. Moreover, we showed that RELB knockdown suppresses the proliferation and migration of HCC cells (Fig. S8 and S9). The interplay between IGF2BP2 and RELB was further confirmed by rescue experiments, in which silencing RELB in IGF2BP2-overexpressing cells abolished the oncogenic effects of IGF2BP2 (Fig. 7). Therefore, RELB appears to play a critical role in promotion of HCC progression by IGF2BP2.

It is reported that the GXXG motif in the KH3 and KH4 domains is essential for the recognition and interaction of IGF2BP2 with target genes [14, 19]. In our study, we found that mutating the GXXG motif in both the KH3 and KH4 domains destabilized RELB mRNA, further reducing its mRNA and protein expression (Fig. 6). These results indicate that IGF2BP2 regulates RELB expression through its KH3 and KH4 domains, which provides deeper insight into the critical functional domains of IGF2BP2 in the regulation of HCC progression. To further investigate the molecular mechanisms by which RELB contributes to HCC progression, we performed RNA-seq in HCC cells transfected with siRELB. KEGG enrichment analysis of differentially expressed genes revealed that TNF signaling pathway was significantly enriched. Notably, the expression of the key gene CCL20 was markedly upregulated upon RELB knockdown (Fig. S12a and S12b). We hypothesized that the non-canonical NF-κB signaling pathway, in which RELB is involved, may contribute to the TNF signaling pathway via CCL20. Further in-depth investigation will be conducted in subsequent work.

Activation of the NF-κB pathway is involved in the pathogenesis of HCC [35]. Several small-molecule compounds targeting the NF-κB pathway have been investigated as potential treatments for HCC [36, 37]. SN52 is a cell-permeable peptide that blocks nuclear import of the RELB:p52 dimer [28, 38]. Previous studies showed that SN52 has therapeutic effects in prostate cancer and breast cancer [22, 28]. In our study, we found that SN52 inhibited HCC cell viability and the nuclear translocation of RELB:p52 dimer (Fig. 8). Moreover, the subcutaneous xenograft tumor model in nude mice showed that, SN52 alone, or combination of SN52 and IGF2BP2 interference, significantly suppressed HCC tumor growth (Fig. 8). These findings suggest that the IGF2BP2-RELB axis may be a novel therapeutic target for HCC, which merits further study.

Nevertheless, several limitations of this study should be acknowledged. First, the analysis was restricted to a cohort of merely 50 clinical specimens; accordingly, our conclusions warrant further substantiation in large‑scale clinical samples. Second, mounting evidence has established that m^6^A methylation reshapes the immune microenvironment of HCC via modulating the expression of immune‑associated mediators, thereby facilitating the malignant progression of HCC [39, 40]. Whether IGF2BP2 likewise exerts regulatory effects on the HCC immune microenvironment remains to be rigorously validated in orthotopic HCC models.

In conclusion, our findings demonstrate that IGF2BP2 plays a critical role in HCC progression by stabilizing RELB mRNA and activating the NF-κB signaling pathway. The significant inhibition of HCC growth through the interference of IGF2BP2 or destabilization of RELB mRNA by SN52 in nude mice provides a potential novel therapeutic target for HCC. Targeting the IGF2BP2-RELB axis may provide a novel therapeutic strategy for HCC.

## Methods

### HCC patient specimens, cells and culture, and lentiviral vectors

Paraffin specimens of HCC tissues and corresponding adjacent tissues were collected from 50 HCC patients who underwent surgery in Nanjing Drum Tower Hospital from 2016 to 2017. The study was approved by the Institutional Ethics Committee of Nanjing Drum Tower Hospital (No. 2024–356-03) and the informed consent was obtained from all patients. This study was performed in line with the principles of the Declaration of Helsinki.

An immortalized human hepatic cell line (LO2) and human HCC cell lines (HepG2, Hep3B, Huh7 and MHCC97H) were conventionally cultured in MEM culture medium (Gibco, Carlsbad, CA, USA) containing 10% fetal bovine serum (ExCell, Suzhou, China) and Penicillin–Streptomycin (100 U/mL penicillin, 100 μg/mL streptomycin; 1% v/v) in a humid environment of 37℃ and 5% CO_2_.

Lentiviral vectors encoding oeIGF2BP2, shIGF2BP2 and oeRELB sequences were purchased from GenePharma (Shanghai, China). Stable single-cell clones overexpressing IGF2BP2 (via oeIGF2BP2 lentiviral) or knocked down for IGF2BP2 (via shIGF2BP2 lentiviral) were selected with puromycin for 14 days post-transfection. siRNA targeting RELB was obtained from GenePharma, and IGF2BP2 mutant plasmids were purchased from GeneChem (Shanghai, China). Transfections of siRNA and plasmids into HCC cells were performed using Lipofectamine 3000 (Invitrogen, Carlsbad, CA, USA) according to the manufacturer’s protocol.

### Immunohistochemical staining (IHC), western blotting and immunofluorescence (IF)

For IHC, the prepared paraffin-embedded tumor tissue sections were incubated with 100-fold diluted anti-IGF2BP2 rabbit polyclonal antibody (BBI, Shanghai, China) at 4℃ overnight and counterstained with hematoxylin after incubation with 5000-fold horseradish peroxidase (HRP)-conjugated goat anti-rabbit IgG (BBI). The staining intensity and area (proportion of positive cell) of each section were separately scored based on a predefined scale. The intensity was scored as 0, 1, 2, and 3 based on the negative, weak, moderate, and strong staining respectively. The staining area was determined based on the proportion of positive cells (0 = 0%, 1 = 1–25%, 2 = 26–50%, 3 = 51–75%, and 4 = 76–100%) [41]. The scores for the staining intensity and area were multiplied to obtain the final IHC score for each section. The highest score was 12 and cut-off score of 5, which corresponded to the threshold exhibiting the optimal balance between sensitivity and specificity in the receiver operating characteristic (ROC) curve. The samples with a final IHC score of ≤ 5 were classified as having low expression of IGF2BP2, while those with a score of > 5 were categorized as exhibiting high expression.

For western blotting, total proteins of HepG2 and Hep3B were each separated by SDS-PAGE and transferred to PVDF membranes (Millipore, Billerica, Massachusetts, USA)), followed by blocking with 5% non-fat milk. The membrane was incubated with the IgG antibody to IGF2BP2 (BBI), RELB (BBI), FLAG (Servicebio, Wuhan, China), and β-actin (BBI) at 4℃ overnight and an HRP-conjugated goat anti-rabbit IgG for 2 h. The signal was visualized by Image Lab software (Bio-Rad, USA).

Hep3B and HepG2 cells were fixed in 4% paraformaldehyde for 15 min and permeated with cold 0.5% Triton X-100 at 22℃ for 20 min, followed by incubation with 500-fold diluted RELB antibody (Servicebio) and HRP-conjugated goat anti-rabbit IgG. DAPI was used to stain the nuclei. The localization of fluorescence was observed with a confocal microscope.

### RNA extraction, quantitative PCR (qPCR) and RNA sequencing

Total RNA was isolated with TRIzol (Invitrogen, Carlsbad, CA, USA) and reversely transcribed into cDNA. qPCR was performed using SYBR Green I Real-Time PCR kit (Vazyme, Nanjing, China) on LightCycler® 480 Real-Time PCR System. The expression levels of each gene were calculated using 2-^ΔΔCT^ method, with β-actin serving as the internal reference for normalization and standardization. The primer sequences are listed in Supplemental Table 1.

RNA sequencing was carried out by LianChuan Genomic Facility (Hangzhou, China) [42]. Data analysis and visualization were performed with R studio.

### Cell proliferation and cell cycle assay

Hep3B and HepG2 cells were plated into 96‑well culture plates at a density of 2000 cells/well. The plate was renewed with fresh medium after 6 h. Cell Counting Kit-8 solution (Beyotime, Shanghai, China) was added to a final concentration of 10% at 0, 24, 48, and 96 h, respectively. The absorbance at 450 nm was measured after incubation for 3 h.

In RELB inhibition groups, the cells were exposed in either SN52 (MCE, Shanghai, China) or its mutant peptides (SN52M) (40 μg/ml) for 48 h. Then cells were incubated with CCK-8 solution as described above.

For the colony formation assay, Hep3B and HepG2 cells were seeded into 35 mm culture dishes (1,000 cells/dish) and cultured under routine culture conditions (37 °C, 5% CO₂) for 14 days. Cell colonies were then fixed using 4% paraformaldehyde solution and stained with 1% crystal violet.

Cell cycle assay was conducted as followings. The dispersed cells (1 × 10^6^) were fixed with 70% ethanol overnight. After washing with cold PBS, the cells were incubated in a buffer containing 0.1 mg/ml RNase and 10 μg/ml propyl iodide (Beyotime, Shanghai, China) for 30 min, followed measurement by FACScan flow cytometry (Becton Dickinson& Co., USA).

### Cell migration and wound healing assay

Hep3B and HepG2 cells were cultured in serum-free medium for 24 h. The dispersed cells (1 × 10^5^) were seeded into the upper chamber of 8.0 μm pore size Transwell inserts (Corning, NY, USA) with 200 μl serum-free medium and 700 μl medium containing 10% FBS was added into the lower chamber. After incubation for 48 h (Hep3B) or 72 h (HepG2), the unmigrated cells were gently wiped off with a cotton swab. The migrated cells were fixed with 75% ethanol for 20 min and stained with 1% crystal violet for 30 min.

Wound healing assay was performed as follows. Hep3B and HepG2 cells were seeded into 6-well plates and cultured until they reached > 90% confluence. A linear scratch was created using a 10 μl pipette tip, and detached cells were gently washed away with PBS. Serum-free medium was then added to the plates to inhibit cell proliferation and promote migration. Images of the wound area were captured at 0, 24, and 48 h post-scratch using an inverted microscope. The scratched area was quantified using ImageJ software, and the wound healing rate was calculated as the percentage reduction in the initial scratched area over time.

### RNA immunoprecipitation assay

The RNA binding protein immunoprecipitation (RIP) was performed using an RIP kit (Absin, Shanghai, China) according to the manufacturer’s protocol. Cells were lysed in RIP lysis buffer containing protease and RNase inhibitors, and lysates were clarified by centrifugation. Magnetic beads were conjugated with either rabbit anti-IGF2BP2 polyclonal antibody (Proteintech, Wuhan, China) or normal rabbit IgG (negative control). The cell lysates were incubated with antibody-conjugated beads at 4 °C overnight to precipitate protein-RNA complexes. After extensive washing, the precipitated RNA was purified and subjected to reverse transcription and qPCR analysis.

### Dual‐luciferase reporter assay

Dual luciferase plasmids were synthesized by Corues Biotchnology (Nanjing, China) and used to identify potential m^6^A recognition sites. HCC cells were uniformly plated in 6‐well plates and transfected with Lipofectamine 3000 transfection reagent (Invitrogen, USA) and then incubated for 2 days. Cells were lysed using reporter gene lysis buffer and centrifuged at 15 000 g for 3 min after incubation. The supernatant was harvested for measurement, and luciferase activity was assayed following the standard protocol provided with the Dual-Luciferase Reporter Gene Assay Kit (Beyotime, China) (Beyotime, China).

### Methylated RNA immunoprecipitation (MeRIP) assay

MeRIP experiments were conducted following the detailed protocols included in the Magna MeRIP m6A Kit supplied by Millipore (Millipore, USA). Total RNA was isolated with Trizol reagent (TaKaRa, Japan) and fragmented into approximately 100-nucleotide segments. The fragmented RNA was then incubated with either an m^6^A-specific antibody (Anti-N6-methyladenosine, ab208577; Abcam, UK) or control mouse IgG. Immunoprecipitated complexes were captured using Protein A/G magnetic beads, followed by purification and storage. After extensive washing, the precipitated RNA was purified and subjected to reverse transcription and qPCR analysis.

### RNA stability assay

Hep3B and HepG2 cells were seeded into 6-well plates at a density of 1 × 10^6^ cells/well. The cells were treated with actinomycin D (Aladdin, Shanghai, China) in a final concentration of 5 μg/ml [43]. Total RNA was extracted at 0, 3 and 6 h, respectively. The relative RNA expression level of RELB was analyzed with qPCR.

### Xenograft model of subcutaneous HCC in nude mice

The animal experiments were approved by the Ethics Animal Committee of Nanjing Drum Tower Hospital (No. DWSY-24089545). The 4-week-old nude mice were randomly divided into different groups (6 mice/group). For subcutaneous tumor model, 5 × 10^6^ shIGF2BP2 cells, which were infected with were injected into the left flank of mice and cells with non-targeting shRNA (shCtrl) were similarly injected into mice to serve as controls. For RELB inhibition groups, SN52 and its mutant peptides (SN52M) (40 μg/mouse) were intraperitoneal injection respectively every other day after 10 days in tumor transplanted. The tumor length (L) and width (W) were measured every 4 days and the tumor volume (V) was calculated by the formula (V = 1/2 × L × W^2^) [44]. All mice were sacrificed after 30 days, and the tumor quality was recorded.

### In vivoxenograft mouse model

Four-week-old male BALB/c nude mice, provided by the Medical Center of Yangzhou University (China), were raised under standardized specific pathogen‑free (SPF) conditions. Each mouse received lateral tail‑vein injection of 2 × 10⁶ cells resuspended in 0.2 mL PBS, with six mice allocated to each group. After 7 weeks, all animals were humanely sacrificed, and liver tissues were promptly harvested. Digital images were captured for subsequent quantification of metastatic tumor nodules.

### Statistical analysis

Statistical analysis was performed with GraphPad Prism software version 9.0.0. At least three independent experiments were performed for each experiment, and the data were presented as mean ± SEM (Standard Error of the Mean). For comparisons between two groups, the student T-test was used. *P* < 0.05 was considered statistically significant.

## Supplementary Information

Supplementary Material 1.

## Data Availability

The original datasets used in this study are available in the Gene Expression Profiling Interactive Analysi (GEPIA) datasets (http://gepia.cancer-pku.cn/) and the Gene Expression Omnibus (GEO) database (https://www.ncbi.nlm.nih.gov/geo). Further inquiries can be directed to the corresponding author.
