# Supplementary material for: The m6A reader IGF2BP2 promotes hepatocellular carcinoma progression via enhancing RELB stability
Source: Mol Biomed. 2026 May 7;7:62. doi: 10.1186/s43556-026-00465-w (PMC13153289; doi:10.1186/s43556-026-00465-w)
Supplement: Supplementary file 1 — Supplementary Material 1. [file 43556_2026_465_MOESM1_ESM.docx]

**Supplementary Figures**

**
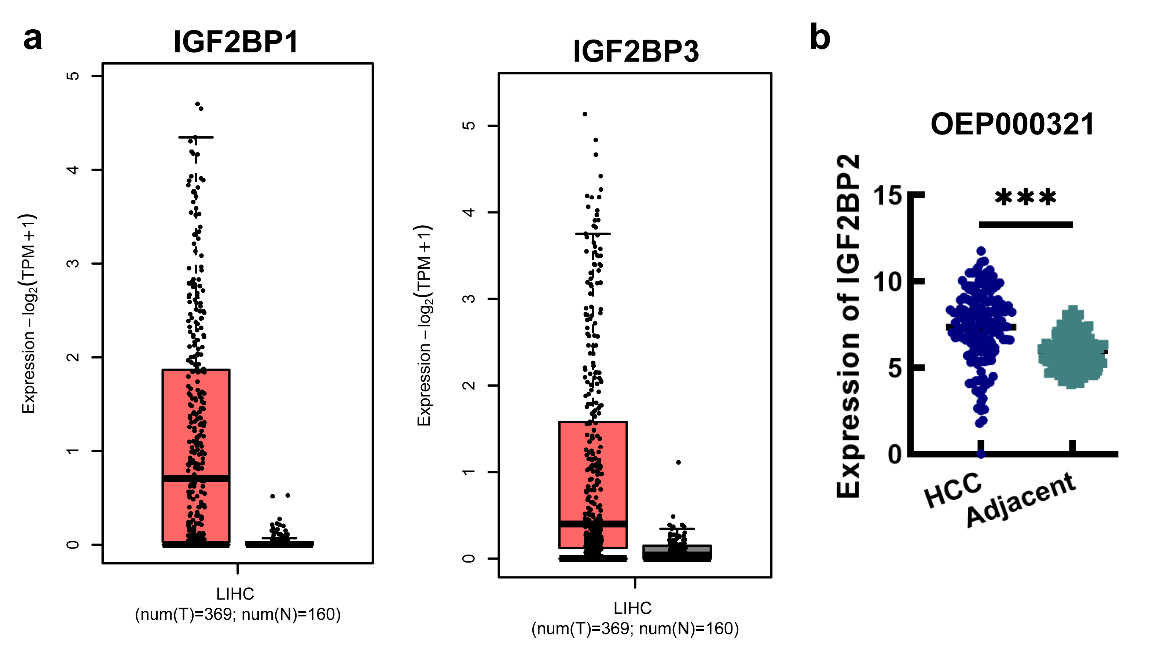
**

**Figure S1. The expression of IGF2BP1/2/3 in public datasets.** (a) The expression of IGF2BP1 and IGF2BP3 in GEPIA dataset. (b)The expression of IGF2BP2 in OEP000321 dataset. Data are mean ± SEM values. ****P*< 0.001.


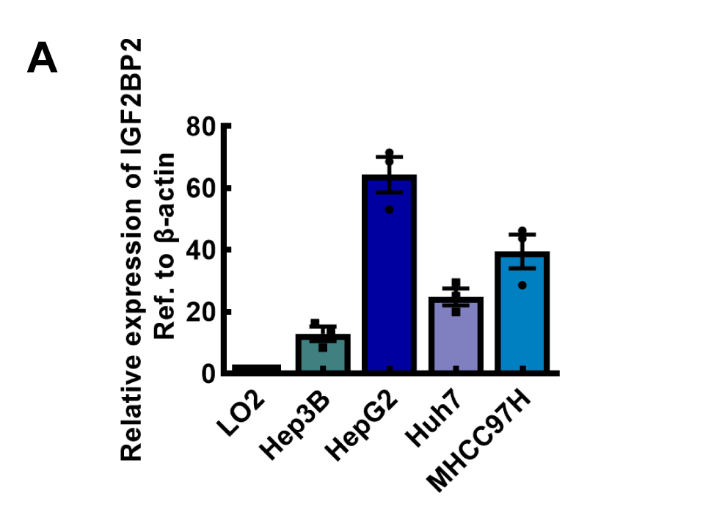


**Figure S2. The relative expression of IGF2BP2 in Hep3B and HepG2 cells.** The mRNA level of IGF2BP2 in LO2, Hep3B, HepG2, Huh7 and MHCC97H were analyzed by RT-qPCR and data were normalized to β-actin. Data are mean ± SEM values (n=3).


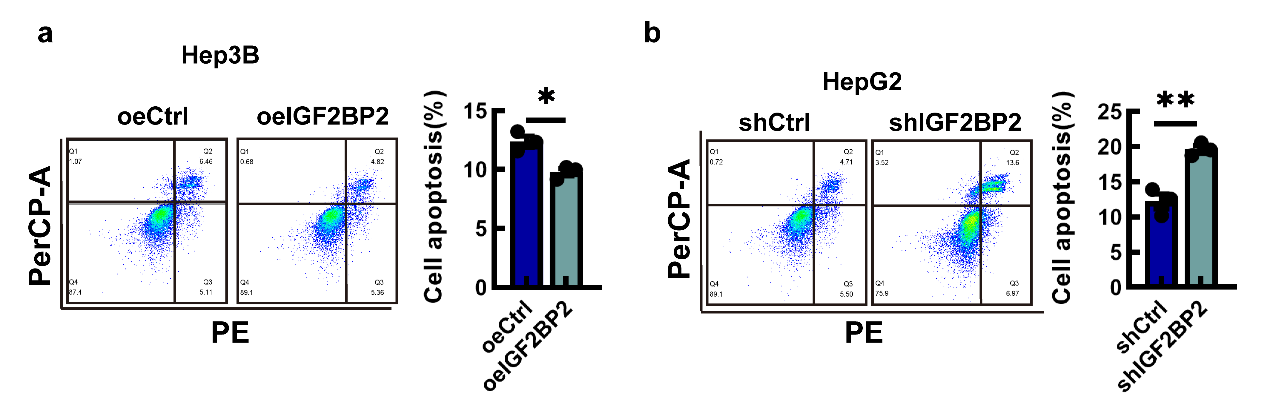


**Figure S3. IGF2BP2 inhibits HCC** **apoptosis.** (a) Apoptosis assays of Hep3B-oeIGF2BP2 cells and Hep3B-oeCtrl cells. (b) Apoptosis assays of HepG2-shIGF2BP2 cells and HepG2-shCtrl cells. Data are mean ± SEM values (n=3). **P*< 0.05, ***P* < 0.01.


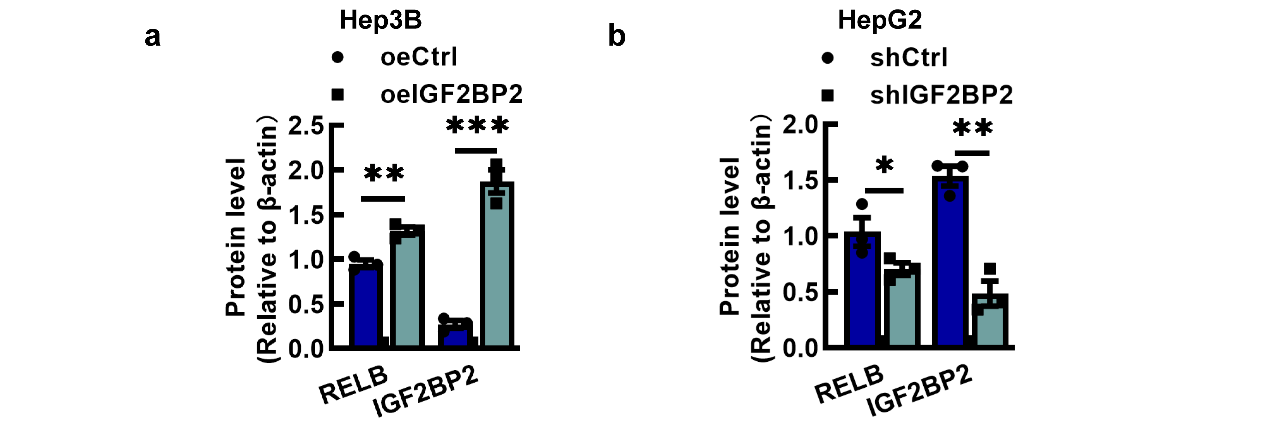


**Figure S4.** **The WB analysis of RELB and IGF2BP2 in HCC cells.** The WB analysis of RELB and IGF2BP2 in Hep3B-oeIGF2BP2 (a), HepG2-shIGF2BP2 (b) and their control cells. Data are mean ± SEM values (n=3). **P*< 0.05, ***P* < 0.01, ****P* < 0.001.


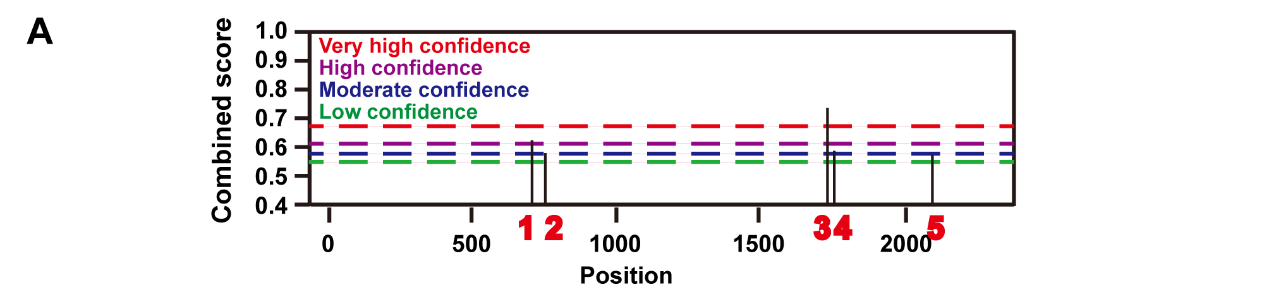


**Figure S5.** The potential binding m⁶A modification sites on RELB mRNA predicted using the SCRAMP tools.


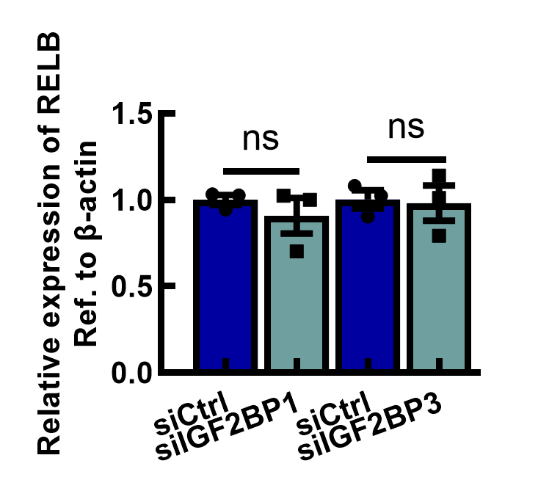


**Figure S6.** RELB expression in HCC cells with siIGF2BP1 and siIGF2BP3. Data are mean ± SEM values (n=3). ns, not significant.


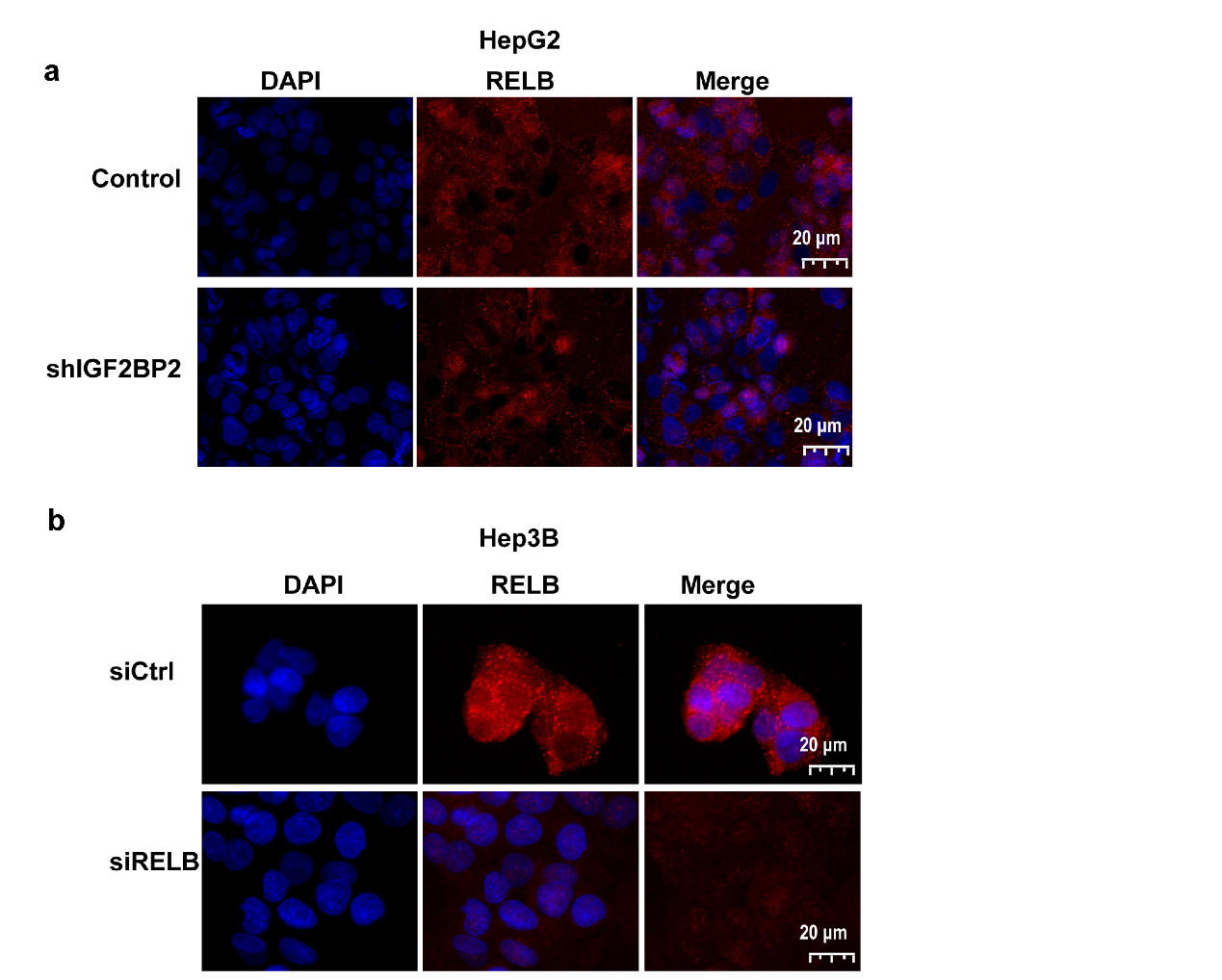


**Figure S7. The Immunofluorescence assay of HepG2-shIGF2BP2 and Hep3B-siRELB cells.** (a) The effect of knockdown IGF2BP2 on RelB:p52 dimer nuclear translocation in HepG2. (b) The effect of knockdown RELB on RelB:p52 dimer nuclear translocation in Hep3B.


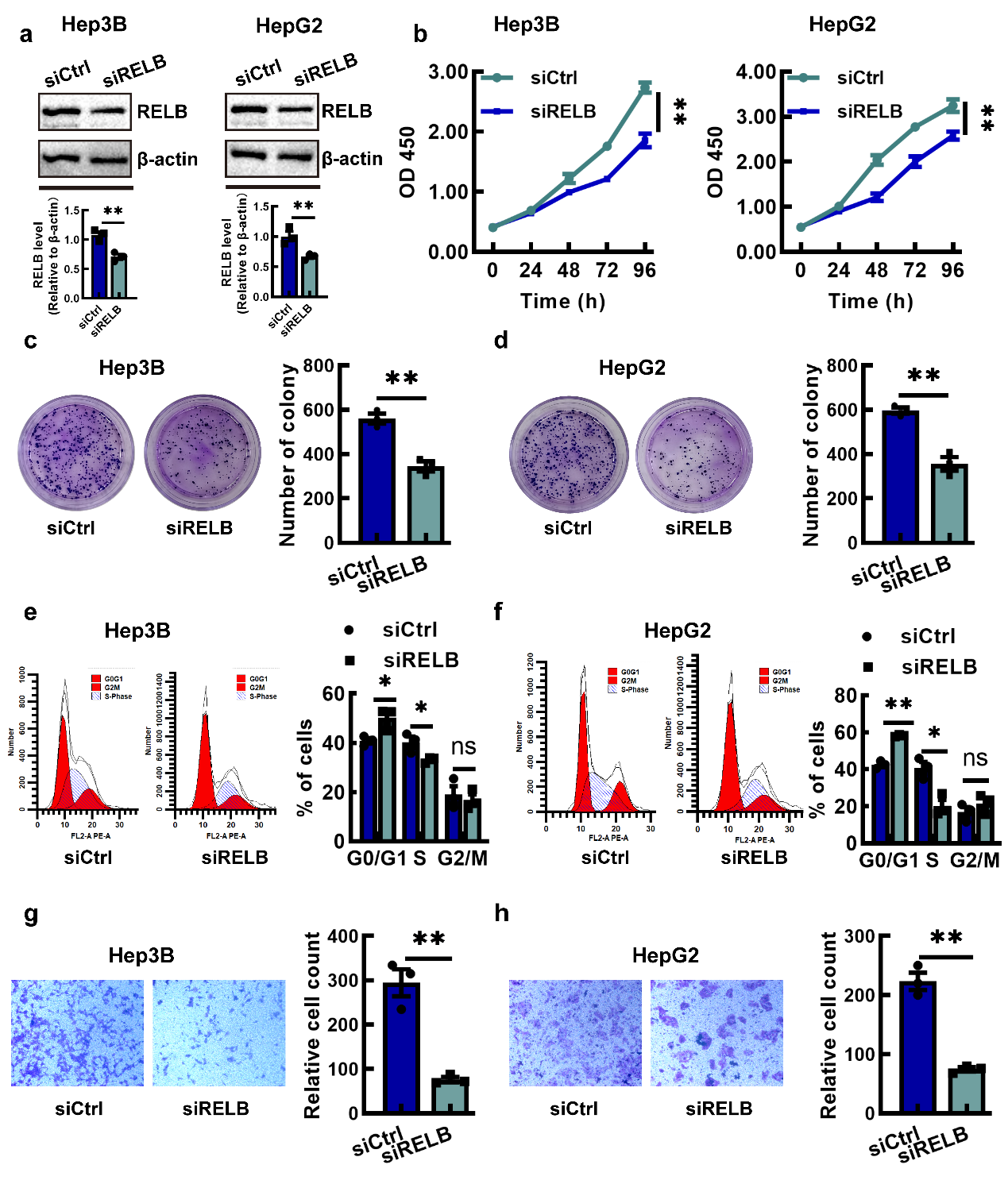


**Figure S8. RELB functions as an oncogene in HCC.** (a) Western blotting analysis of RELB in Hep3B and HepG2 cells with silencing RELB. (b-d) CCK-8 assays and colony formation assays of Hep3B and HepG2 cells with silencing RELB. (e, f) Cell cycle assays of Hep3B and HepG2 cells after silencing RELB. (g, h) Cell migration assays of Hep3B and HepG2 cells with RELB knockdown. (Scale bar = 200 μm). Data are mean ± SEM values (n=3). **P* < 0.05, ***P* < 0.01.


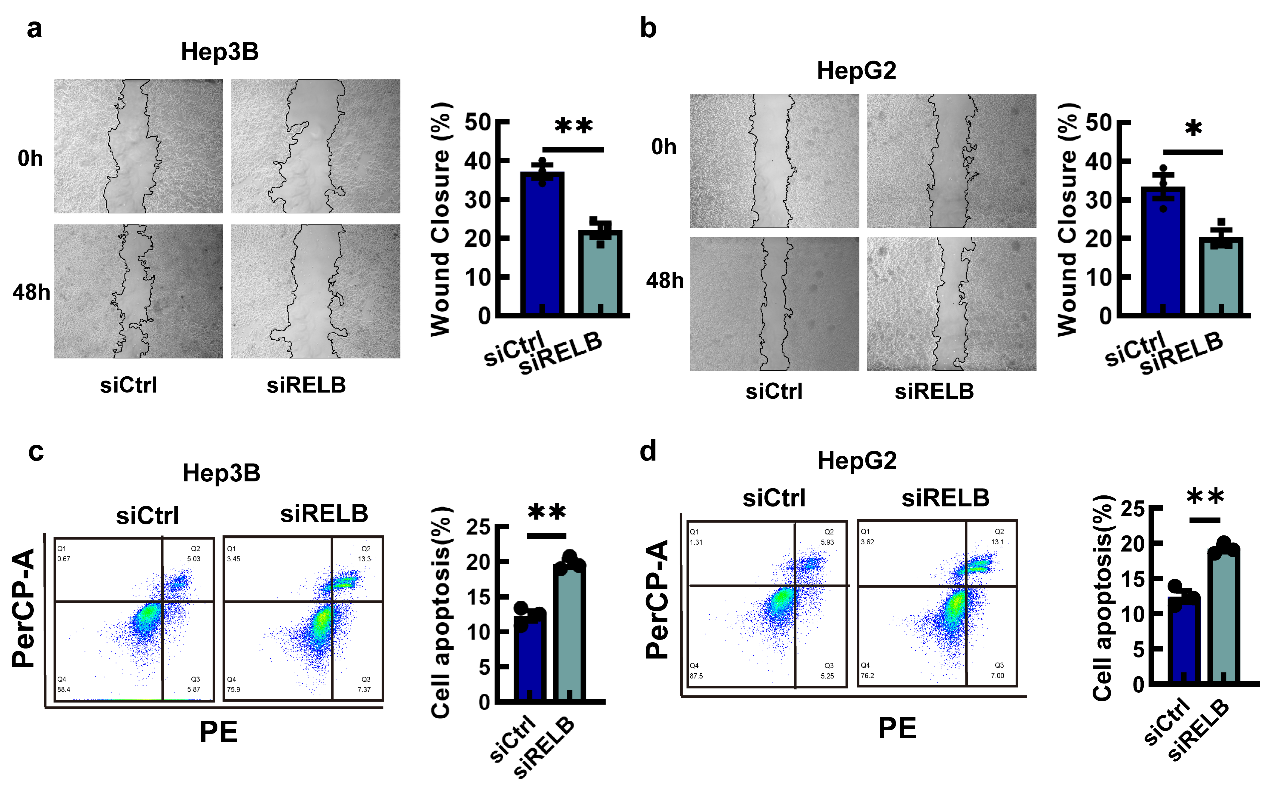


**Figure S9. The effect of RELB on cell migration and apoptosis.** Wound healing assays were used to show RELB inhibition on cell migration ability in Hep3B (a) and HepG2 cells (b). The apoptosis assays were used to show RELB inhibition on cell apoptosis in Hep3B (c) and HepG2 cells (d). Data are mean ± SEM values (n=3). **P* < 0.05, ***P* < 0.01.


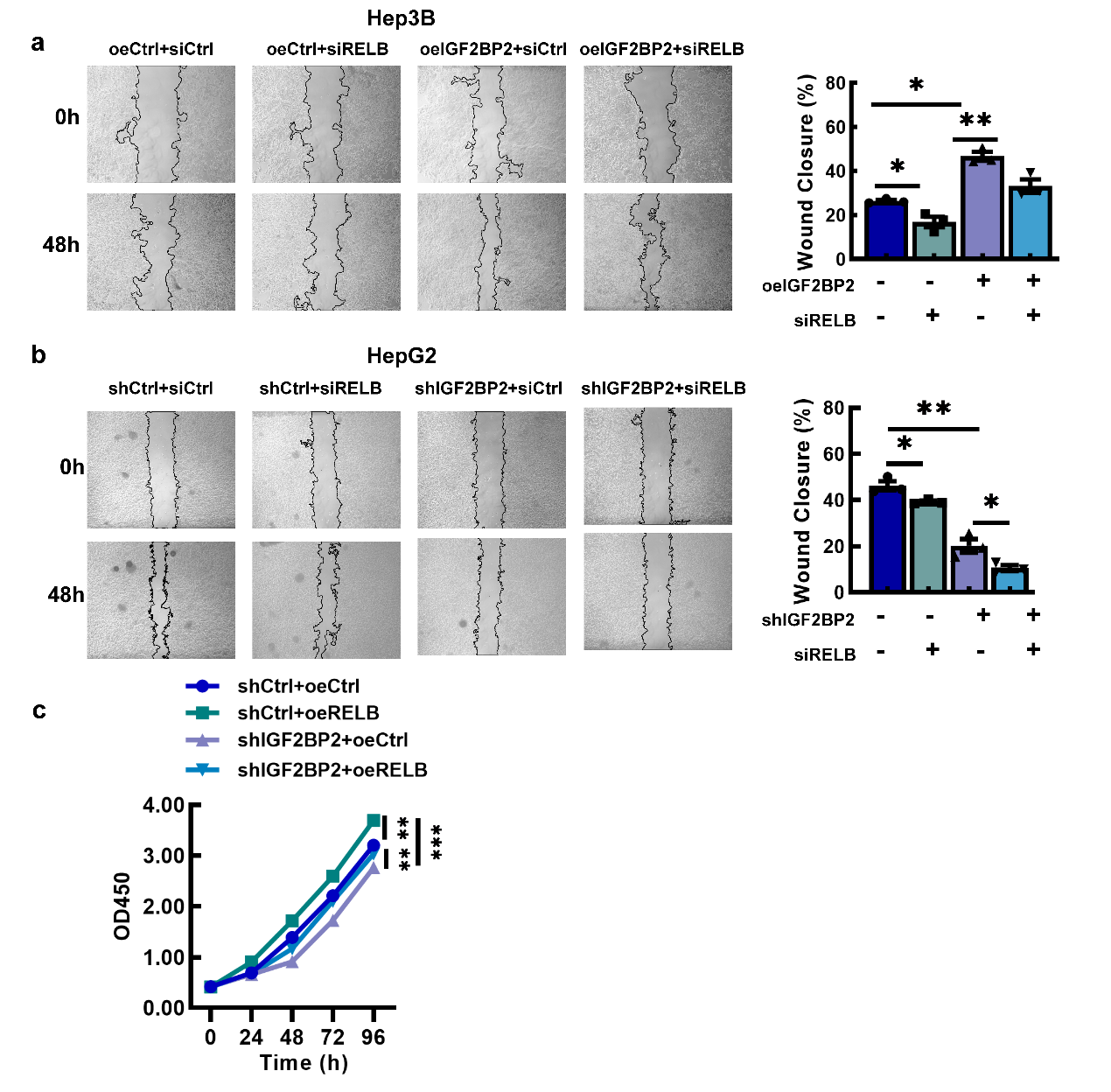


**Figure S10. IGF2BP2 enhanced HCC cell migration via RELB.** The effect of RELB on cell migration ability in Hep3B cells with IGF2BP2 overexpression (a) and HepG2 cells with IGF2BP2 knockdown (b) were validated by wound healing assays. (c) RELB overexpression reversed IGF2BP2 knockdown-induced suppression of HCC cell proliferation. Data are mean ± SEM values.(n=3) **P* < 0.05, ***P* < 0.01, ****P* < 0.001.


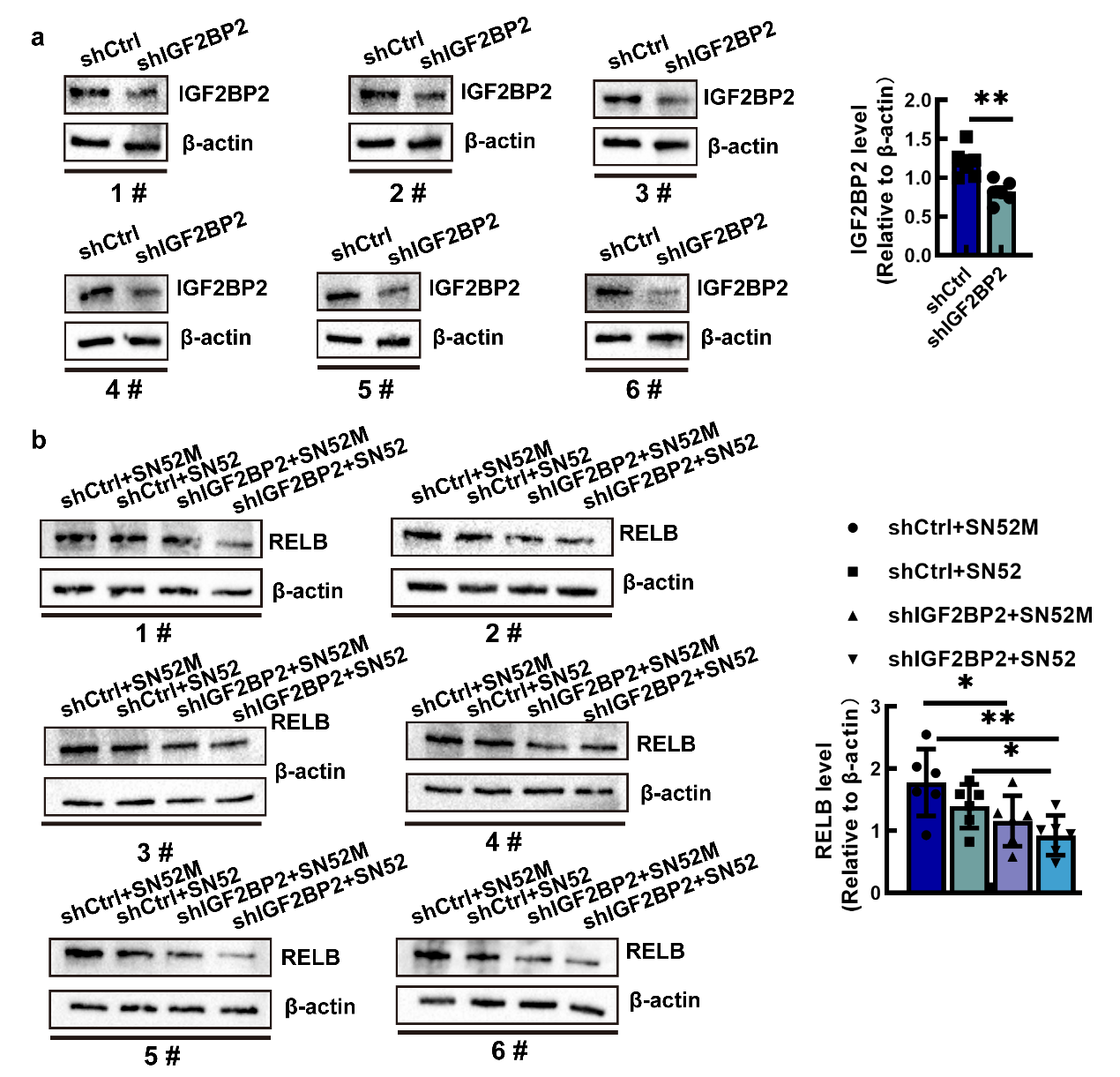


**Figure S11. WB analysis of protein levels in tumor tissue samples.** (a) WB analysis of IGF2BP2 levels in tumor tissue samples in Fig 2G. (b)WB analysis of RELB levels in tumor tissue samples in Fig 9D. Data are mean ± SEM values (n=3). **P* < 0.05, ***P* < 0.01.

**
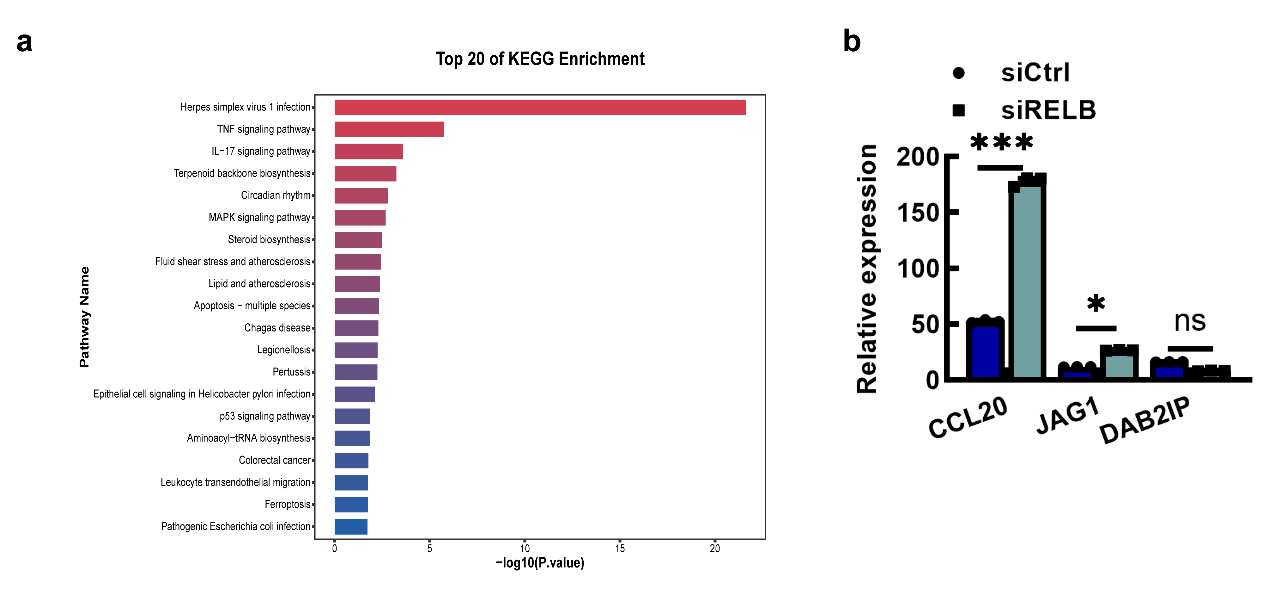
**

**Figure S12. RNA-seq analysis of Hep3B cells with siRELB.** (a)KEGG enrichment analysis of the differently expression genes in Hep3B-siRELB cells. (b) The expression of CCL20, JAG1 and DAB2IP in Hep3B-siRELB cells. Data are mean ± SEM values. **P* < 0.05, ****P* < 0.001, ns, not significant.

**Supplementary Tables**

**Table S1 Primers used for RT-qPCR**

| Real-time PCR | 5’-3’ |
| --- | --- |
| ACTIN-F | GTCATTCCAAATATGAGATGCGT |
| ACTIN-R | GCTATCACCTCCCCTGTGTG |
| IGF2BP2-F | GTAAAGTGGAATTGCATGGGA |
| IGF2BP2-R | CAAAAGTCCATCCAACACCTC |
| RELB-F | AAGACUGCACCGACGGCAUCU |
| RELB-R | AGAUGCCGUCGGUGCAGUCUU |
